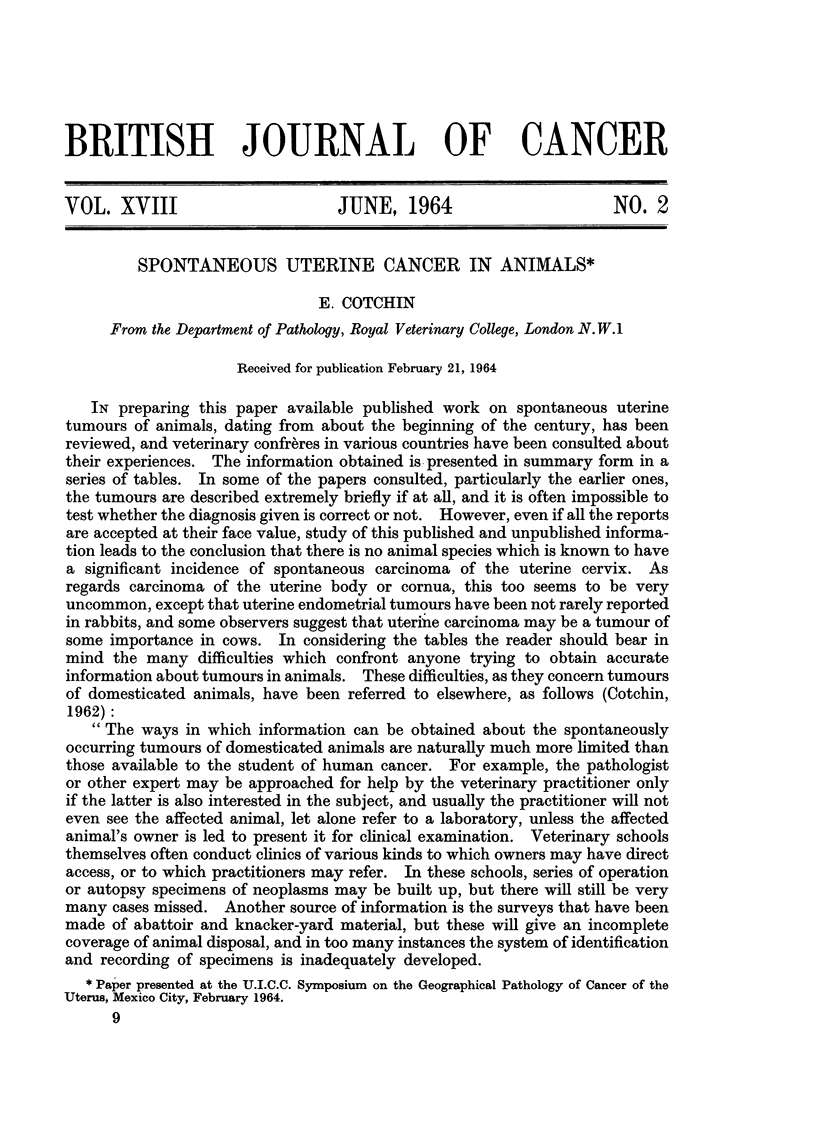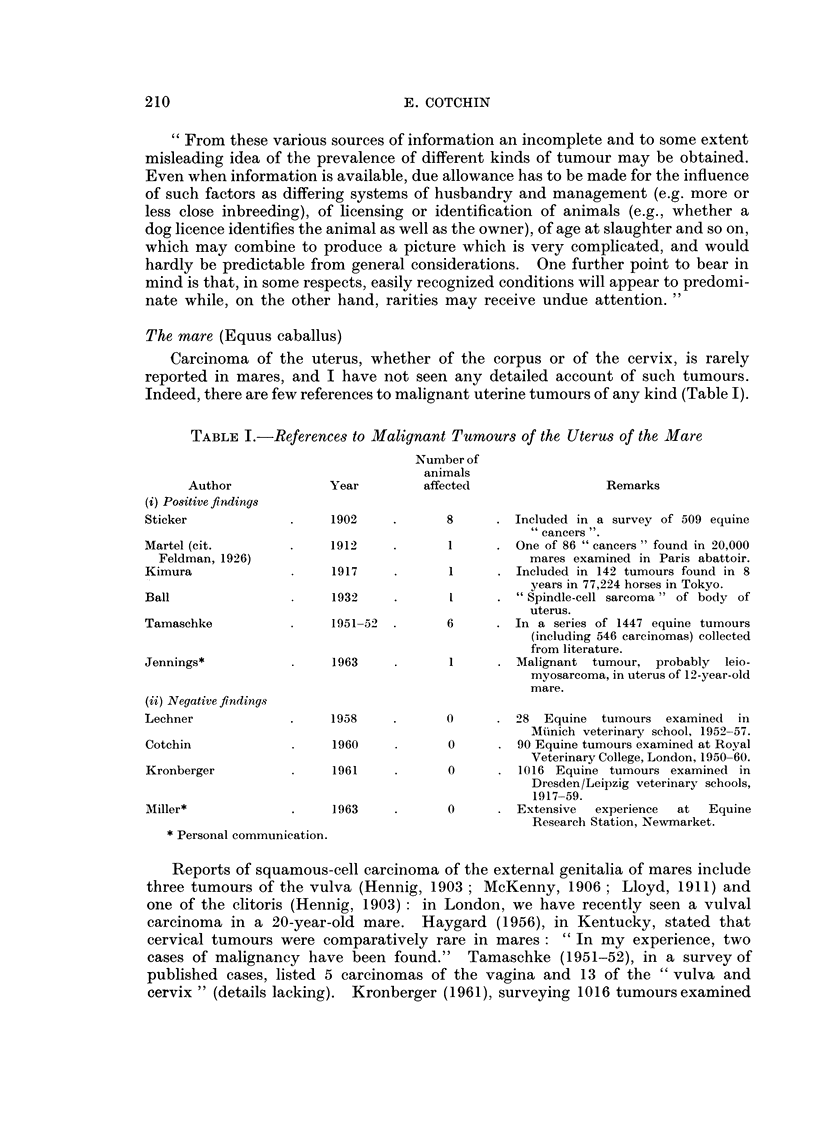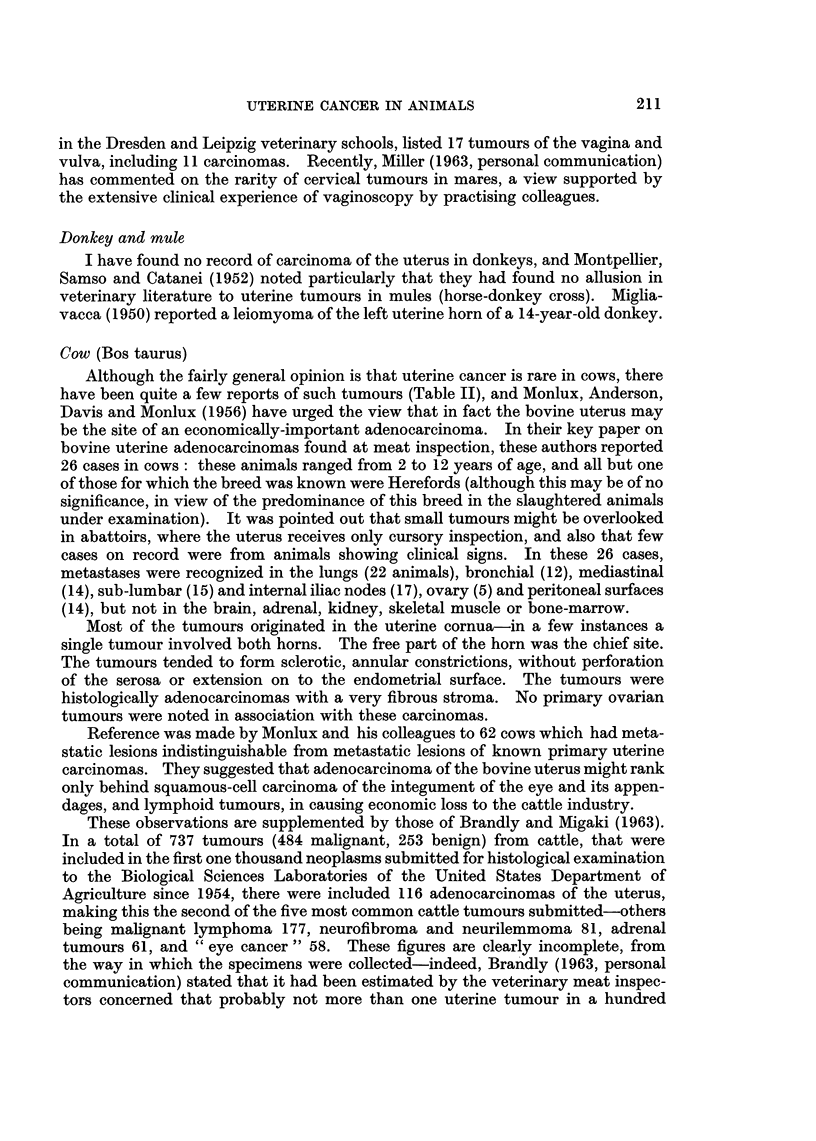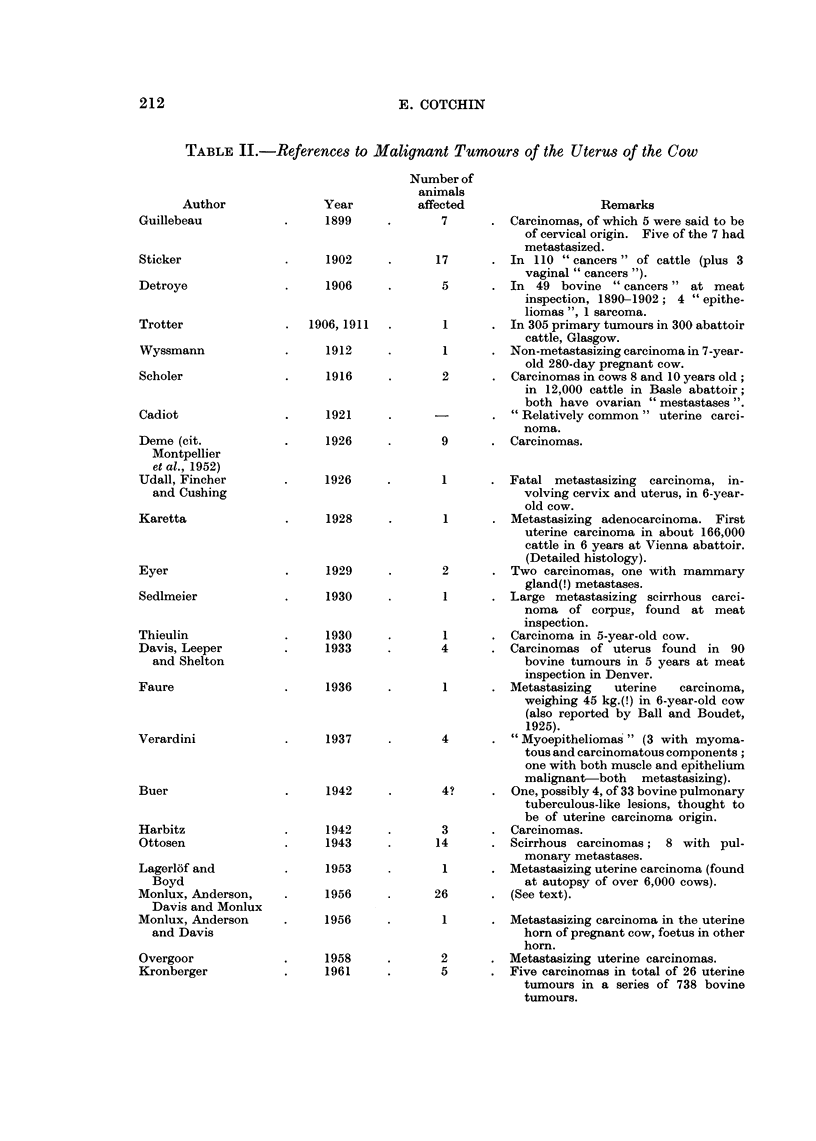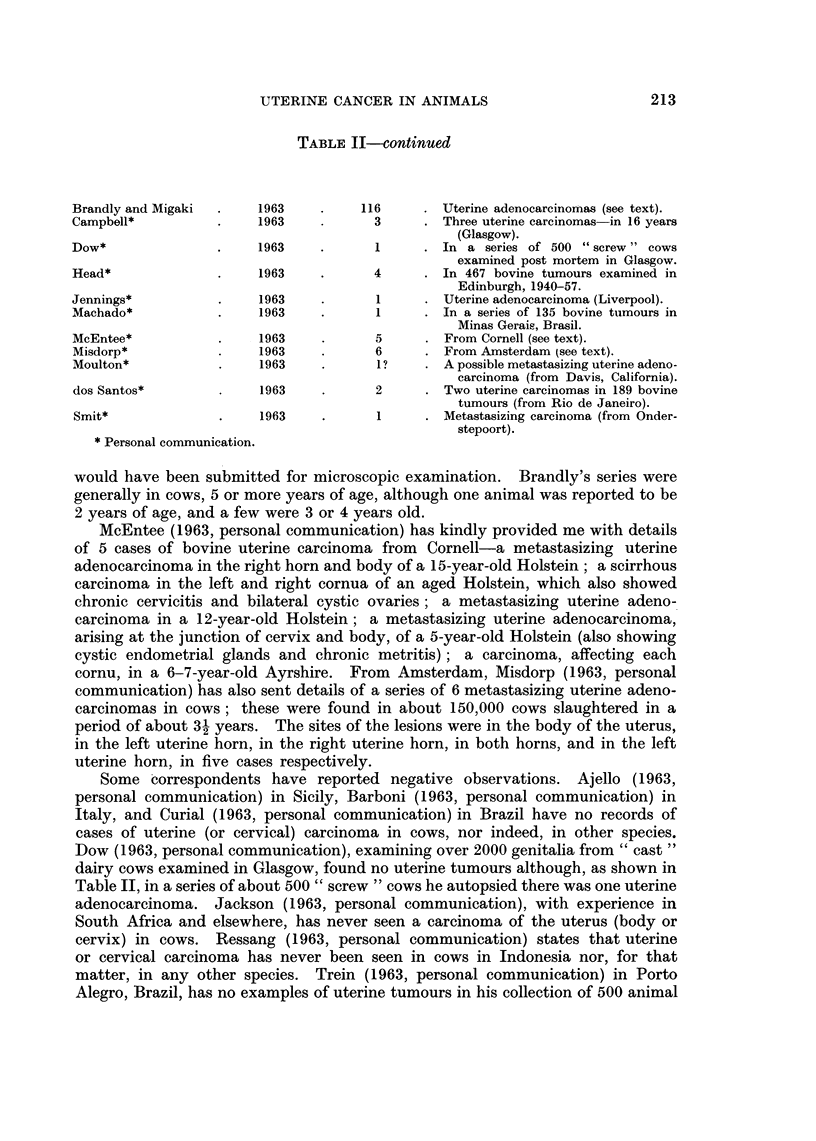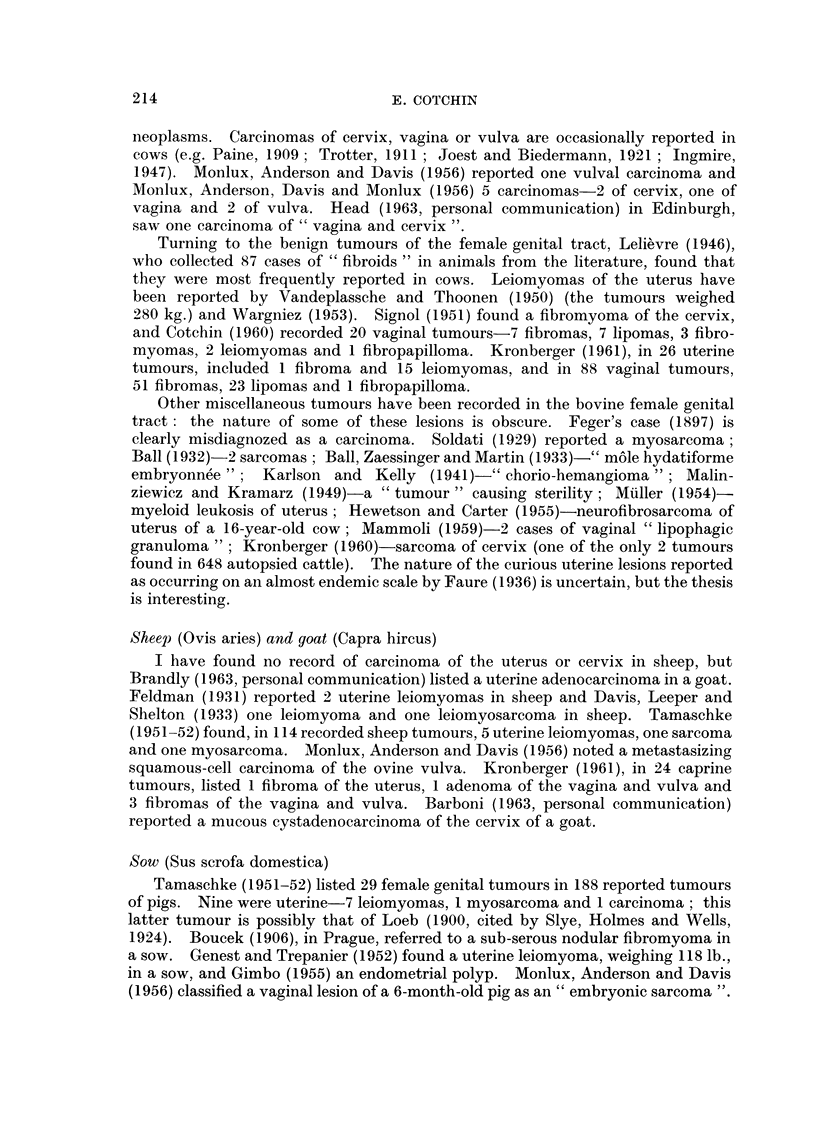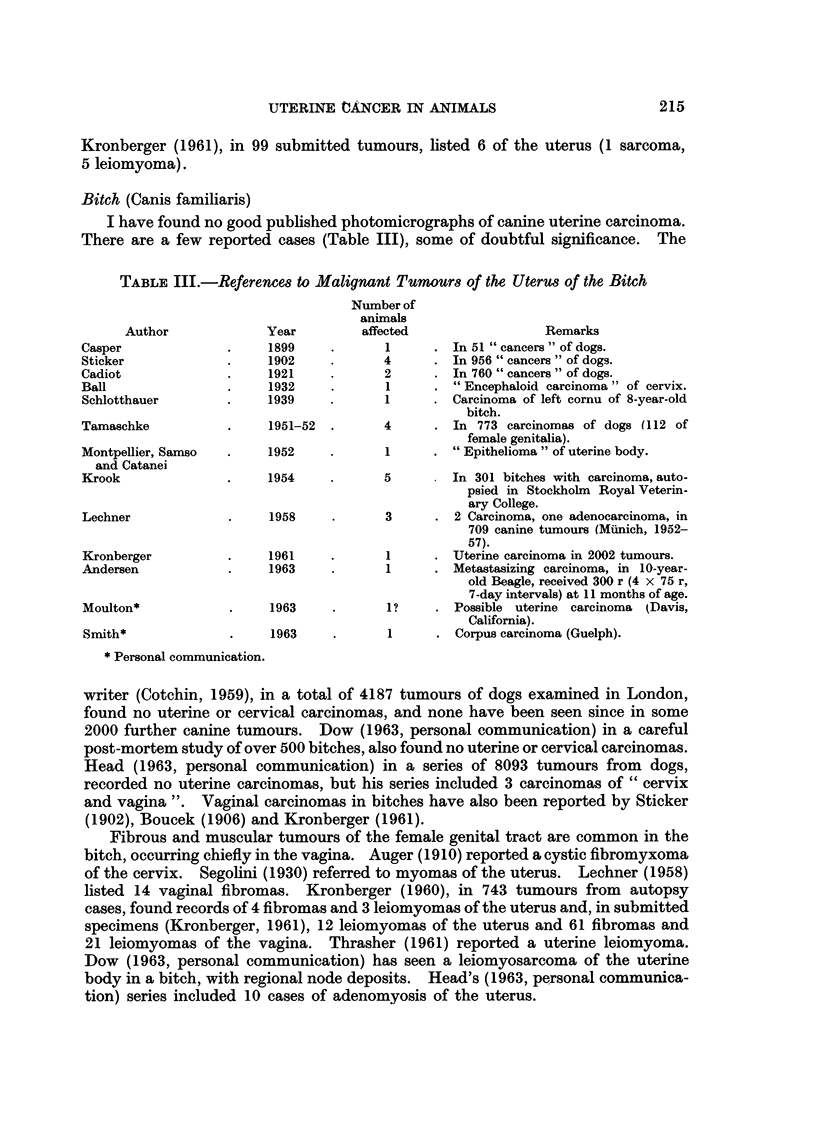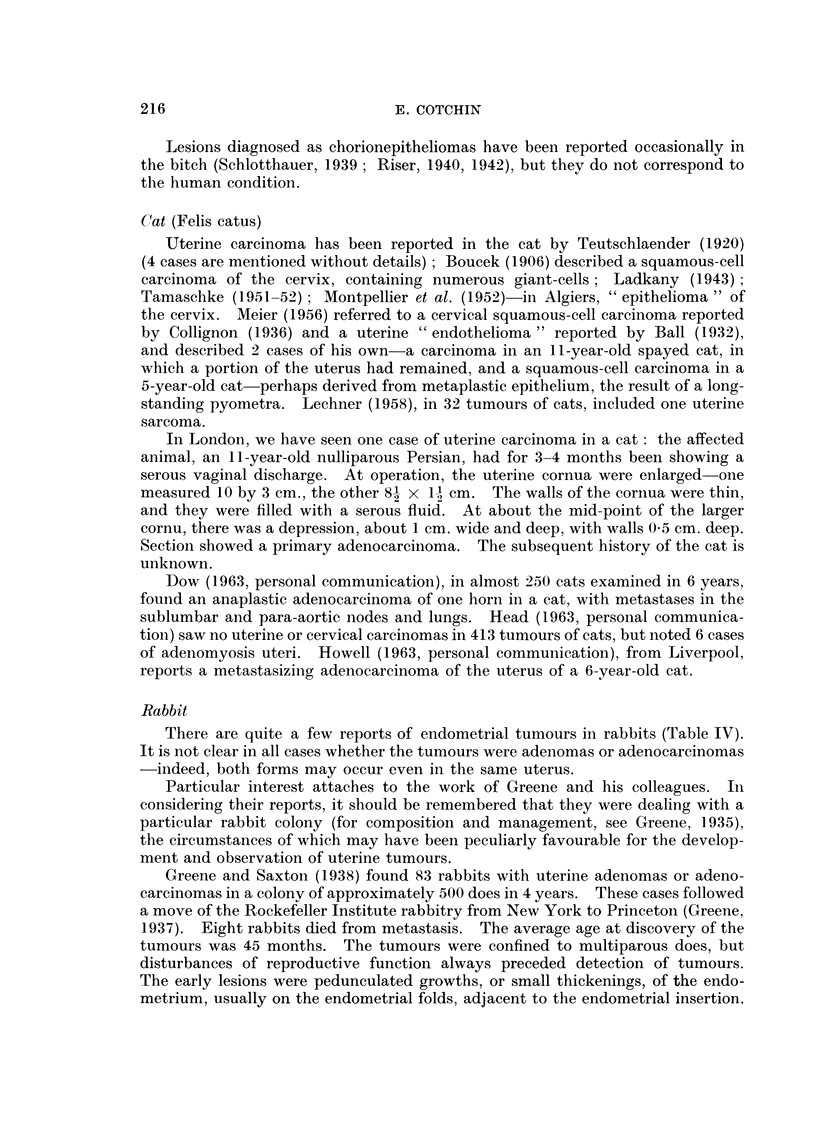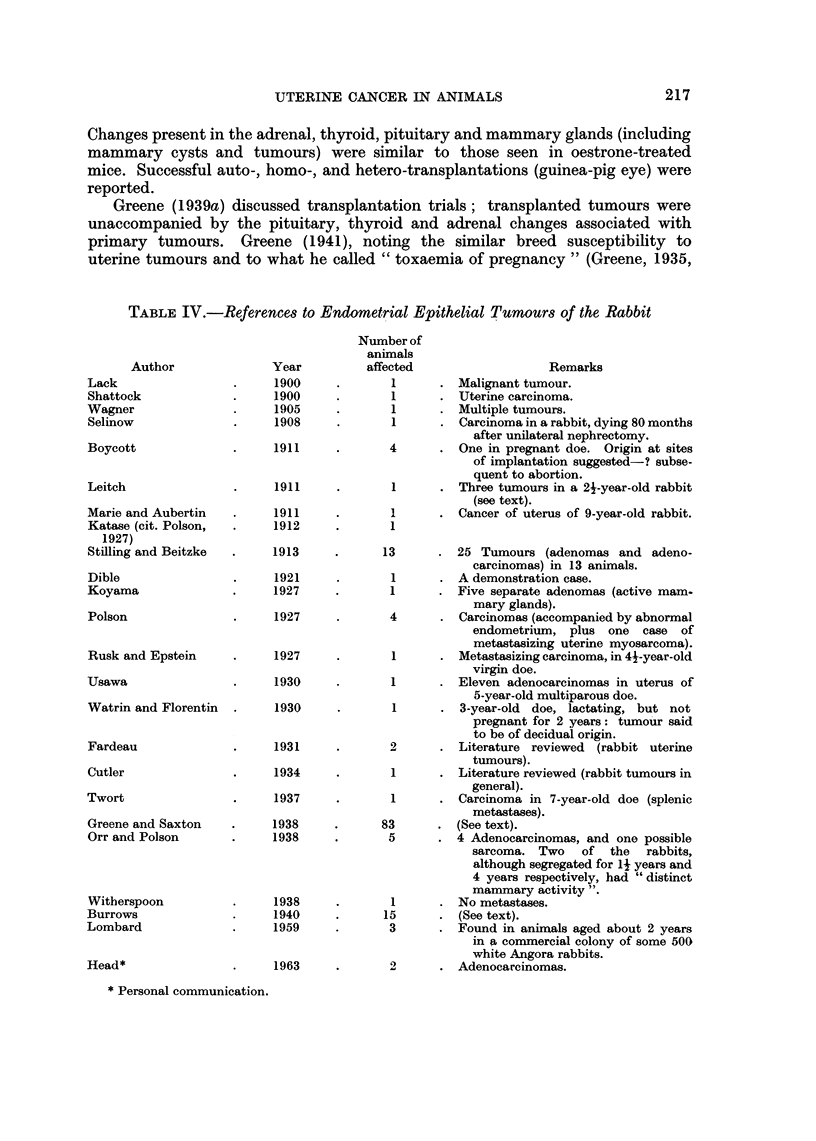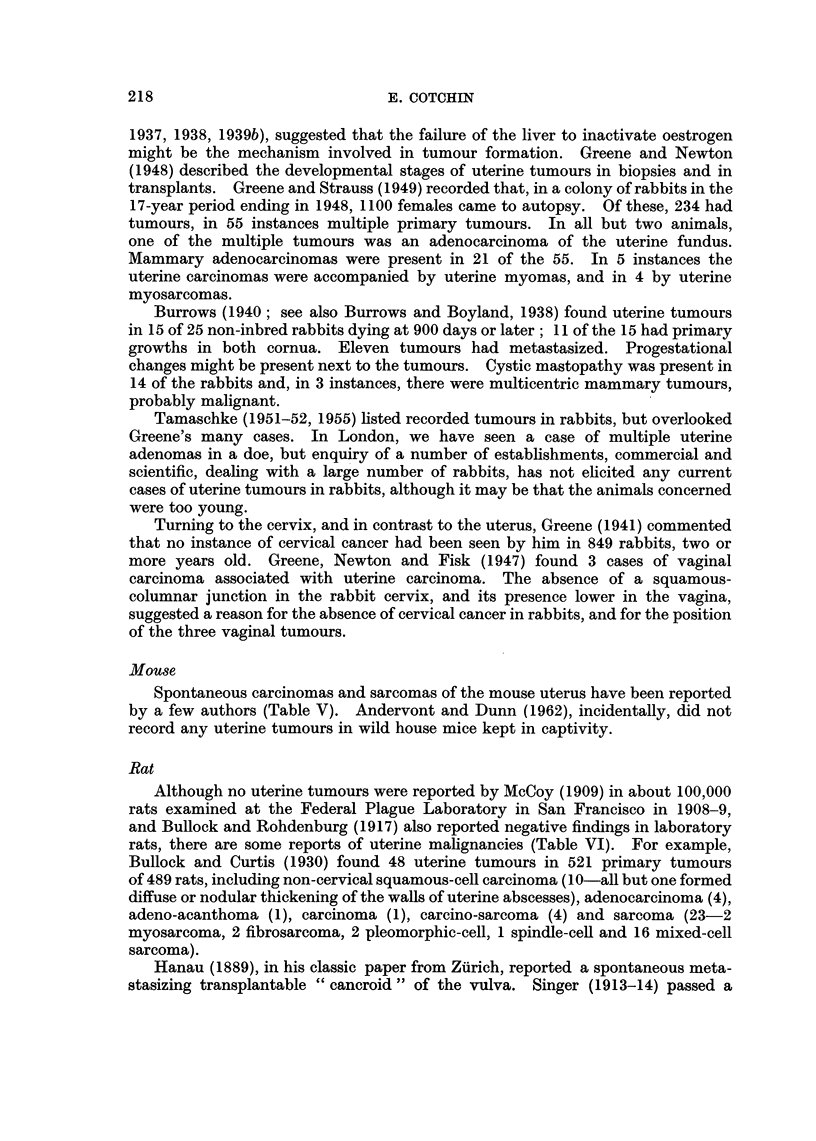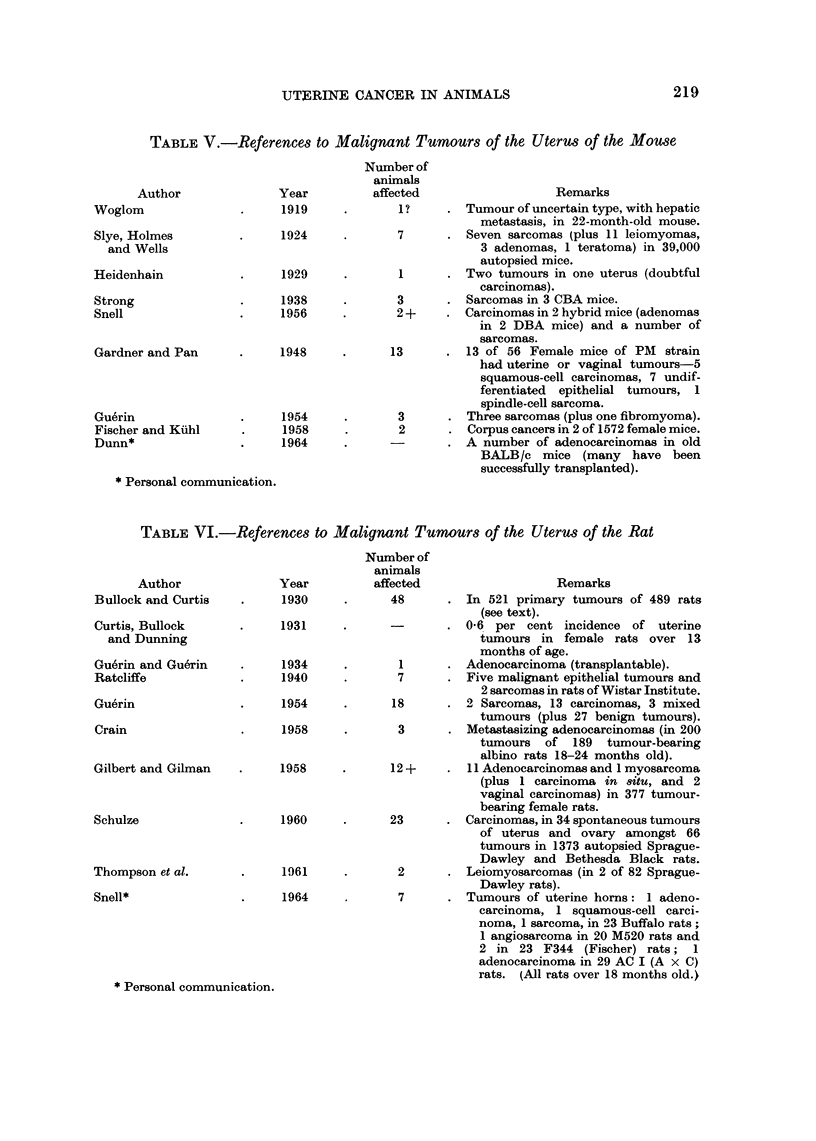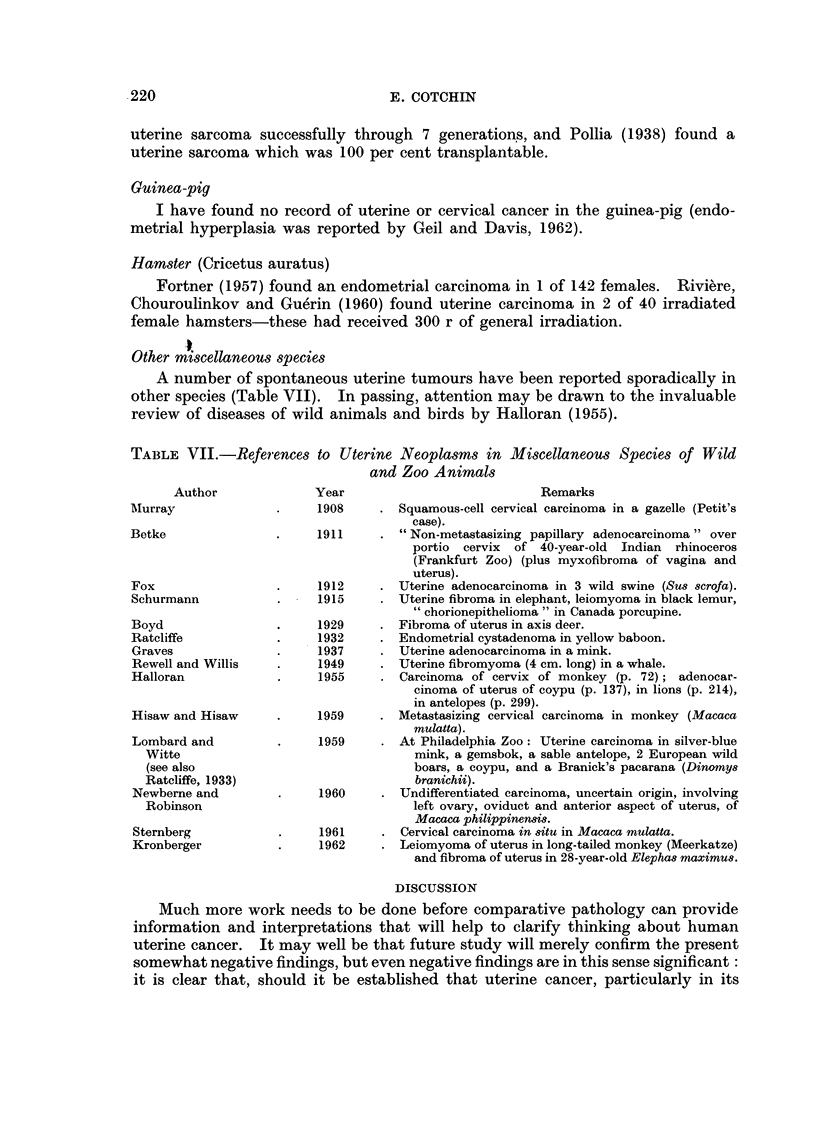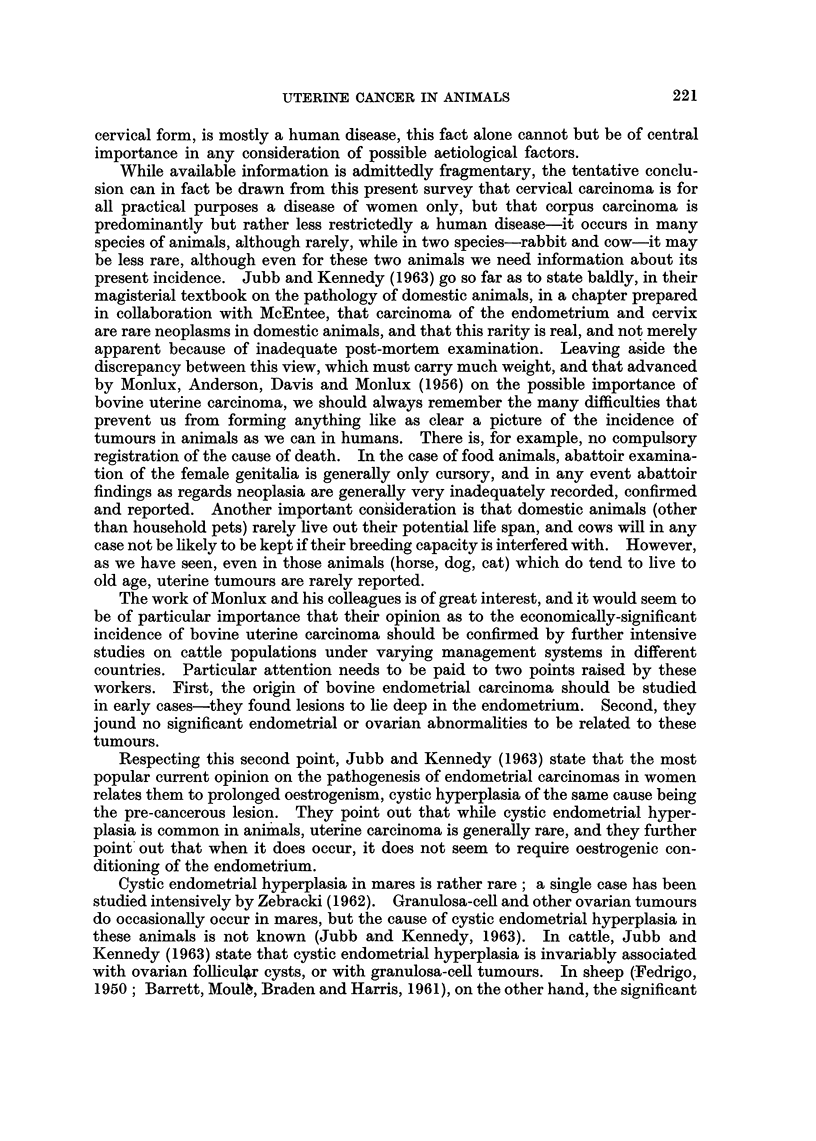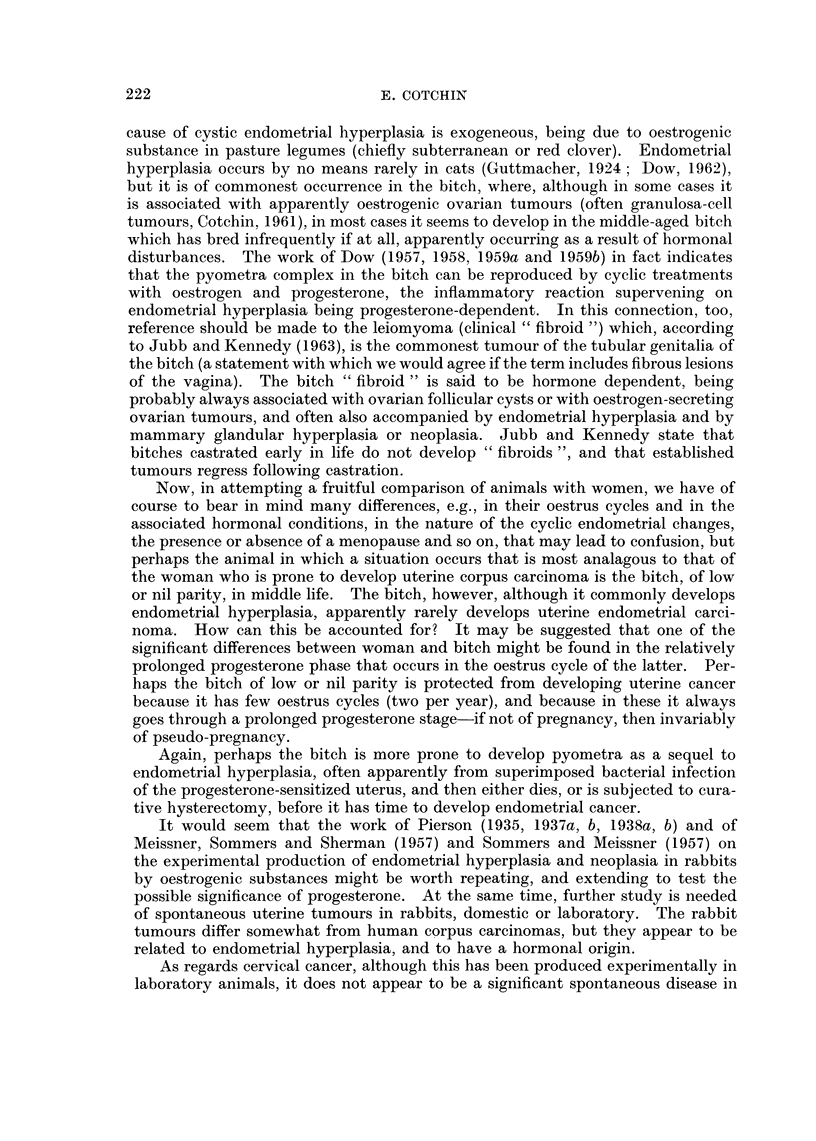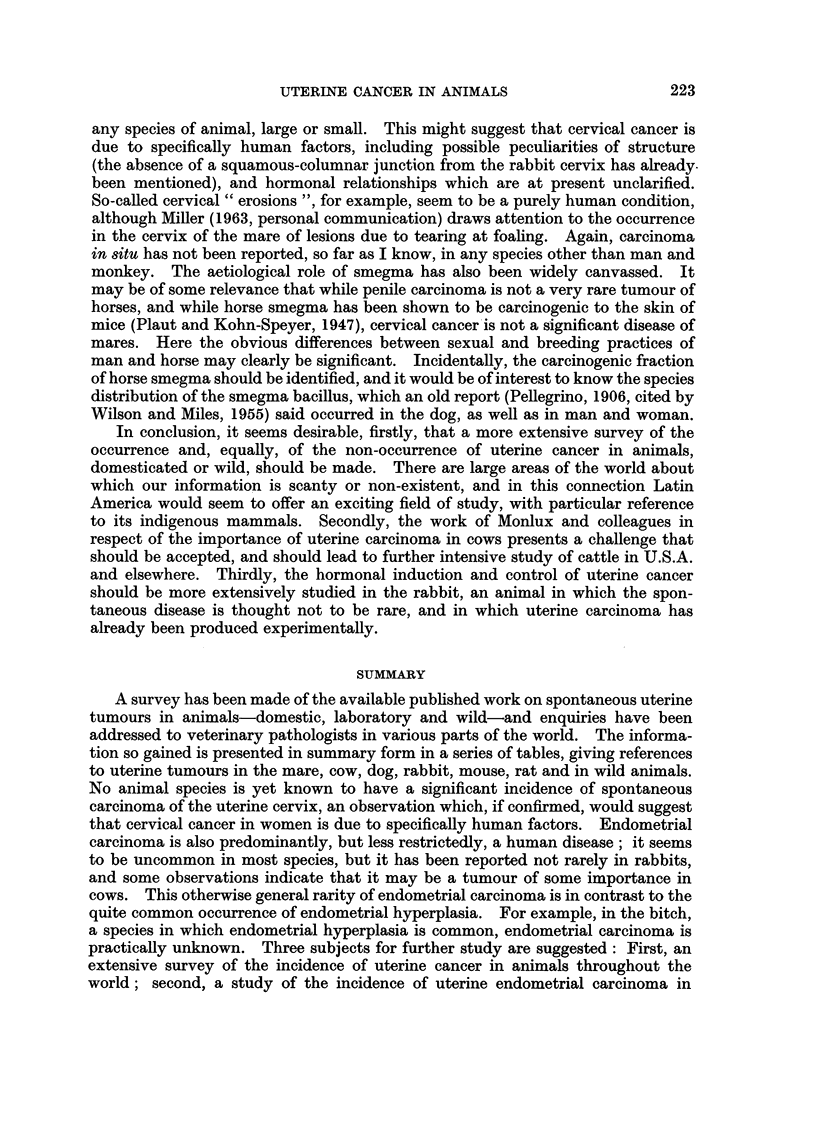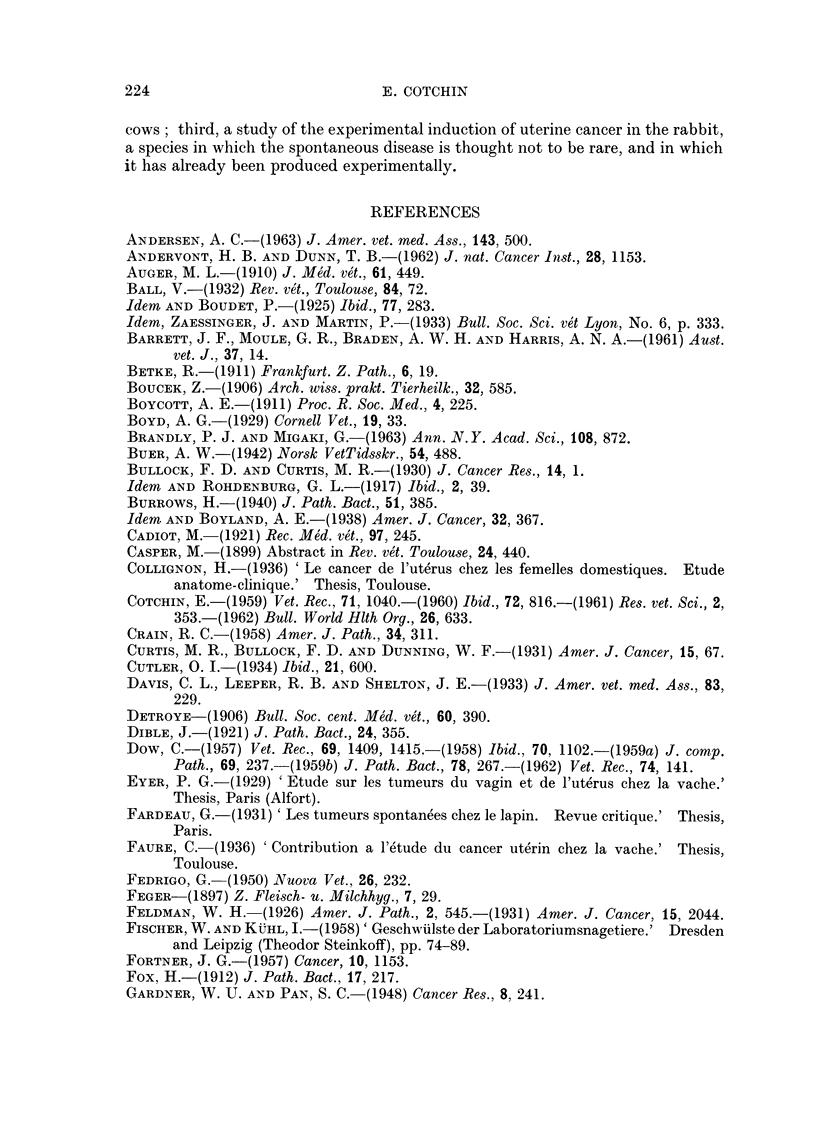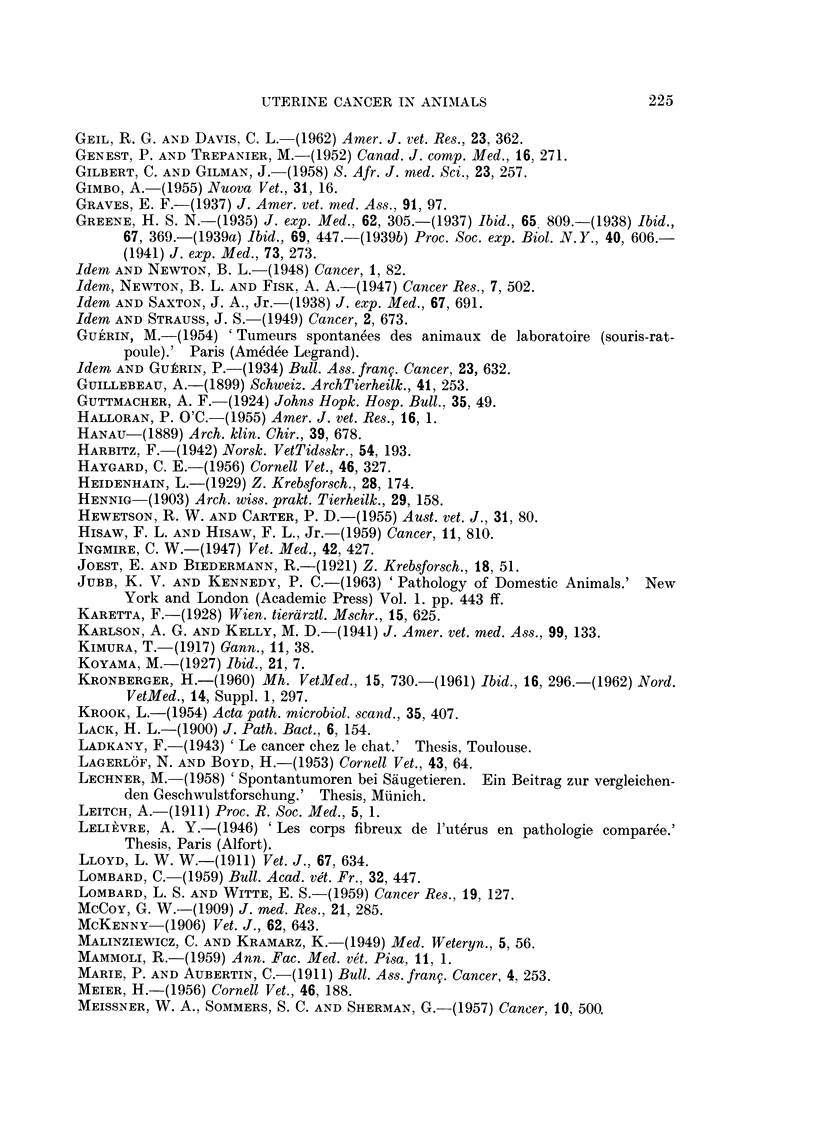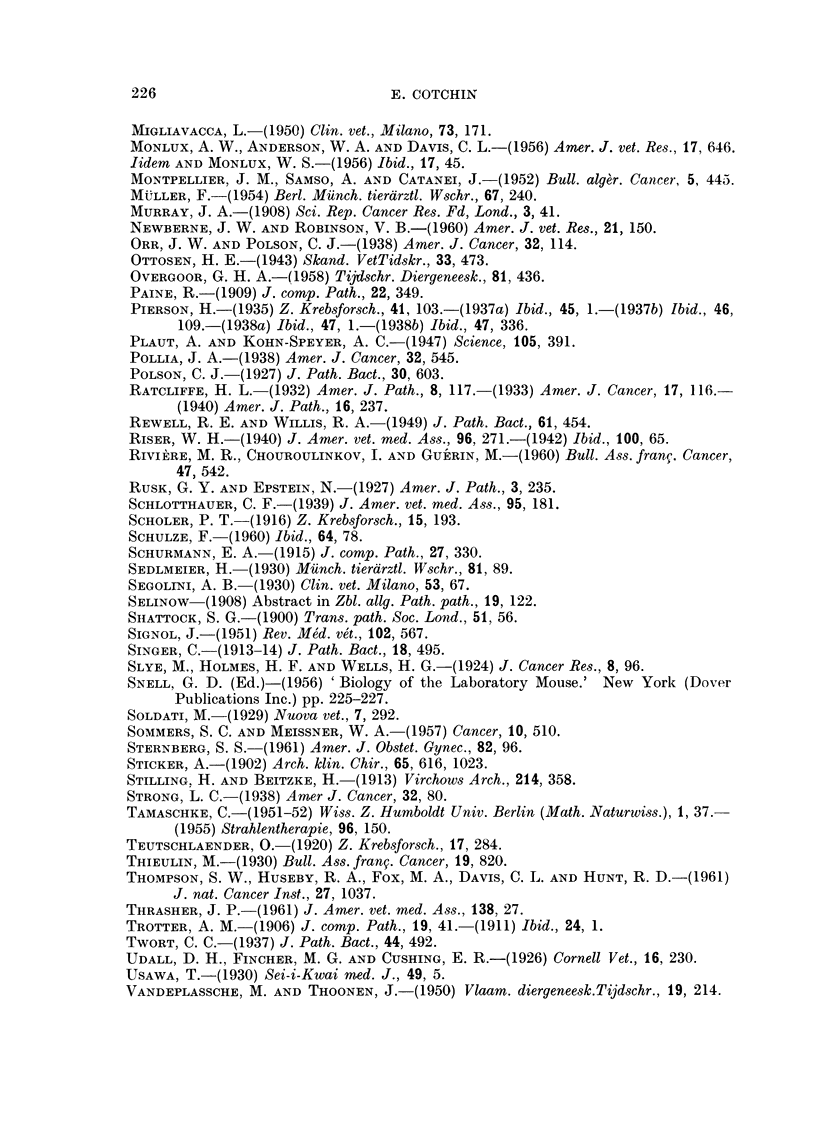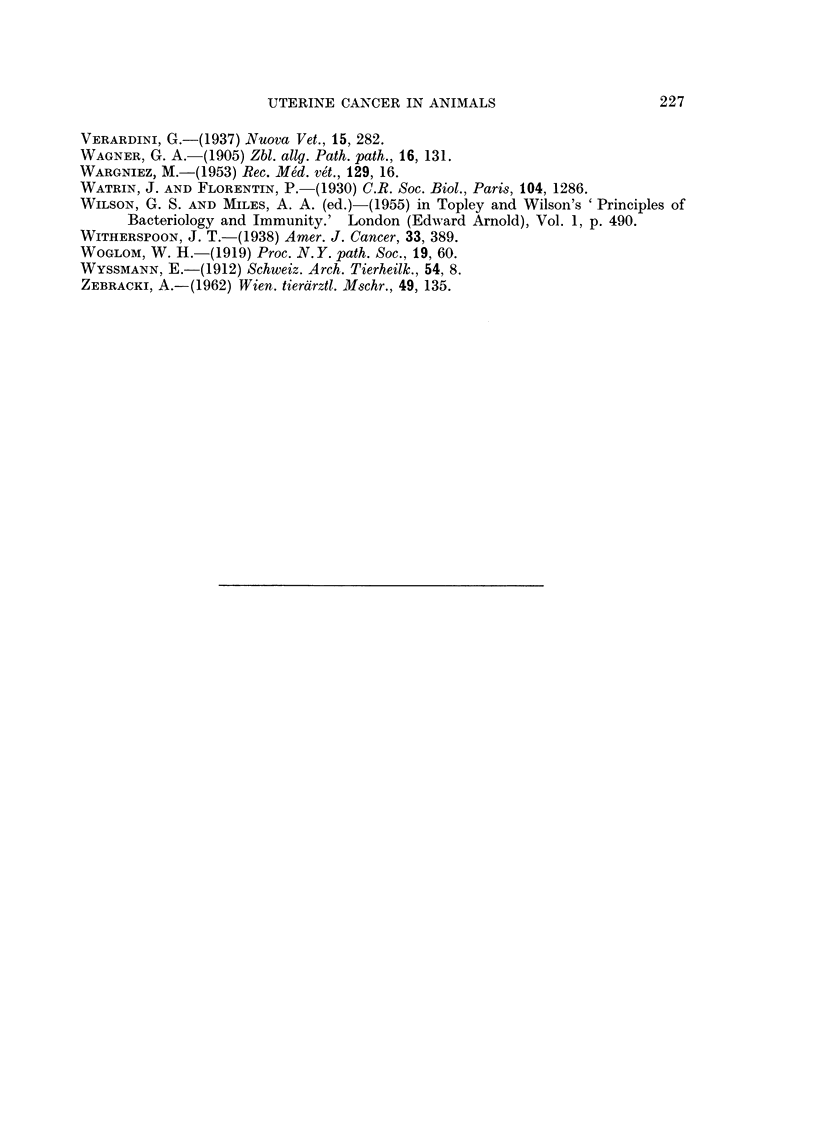# Spontaneous Uterine Cancer in Animals*

**DOI:** 10.1038/bjc.1964.23

**Published:** 1964-06

**Authors:** E. Cotchin


BRITISH JOURNAL OF CANCER

VOL. XVIII JUNE, 1964 NO. 2

### SPONTANEOUS UTERINE CANCER IN ANIMALS*

E. COTCHIN

*From the Department of Pathology, Royal Veterinary College, London N.W.1*

Received for publication February 21, 1964

IN preparing this paper available published work on spontaneous uterine tumours of animals, dating from about the beginning of the century, has been reviewed, and veterinary confrères in various countries have been consulted about their experiences. The information obtained is presented in summary form in a series of tables. In some of the papers consulted, particularly the earlier ones, the tumours are described extremely briefly if at all, and it is often impossible to test whether the diagnosis given is correct or not. However, even if all the reports are accepted at their face value, study of this published and unpublished information leads to the conclusion that there is no animal species which is known to have a significant incidence of spontaneous carcinoma of the uterine cervix. As regards carcinoma of the uterine body or cornua, this too seems to be very uncommon, except that uterine endometrial tumours have been not rarely reported in rabbits, and some observers suggest that uterine carcinoma may be a tumour of some importance in cows. In considering the tables the reader should bear in mind the many difficulties which confront anyone trying to obtain accurate information about tumours in animals. These difficulties, as they concern tumours of domesticated animals, have been referred to elsewhere, as follows (Cotchin, 1962):

" The ways in which information can be obtained about the spontaneously occurring tumours of domesticated animals are naturally much more limited than those available to the student of human cancer. For example, the pathologist or other expert may be approached for help by the veterinary practitioner only if the latter is also interested in the subject, and usually the practitioner will not even see the affected animal, let alone refer to a laboratory, unless the affected animal's owner is led to present it for clinical examination. Veterinary schools themselves often conduct clinics of various kinds to which owners may have direct access, or to which practitioners may refer. In these schools, series of operation or autopsy specimens of neoplasms may be built up, but there will still be very many cases missed. Another source of information is the surveys that have been made of abattoir and knacker-yard material, but these will give an incomplete coverage of animal disposal, and in too many instances the system of identification and recording of specimens is inadequately developed.

* Paper presented at the U.I.C.C. Symposium on the Geographical Pathology of Cancer of the Uterus, Mexico City, February 1964.

" From these various sources of information an incomplete and to some extent misleading idea of the prevalence of different kinds of tumour may be obtained. Even when information is available, due allowance has to be made for the influence of such factors as differing systems of husbandry and management (e.g. more or less close inbreeding), of licensing or identification of animals (e.g., whether a dog licence identifies the animal as well as the owner), of age at slaughter and so on, which may combine to produce a picture which is very complicated, and would hardly be predictable from general considerations. One further point to bear in mind is that, in some respects, easily recognized conditions will appear to predominate while, on the other hand, rarities may receive undue attention. "

*The mare* (Equus caballus)

Carcinoma of the uterus, whether of the corpus or of the cervix, is rarely reported in mares, and I have not seen any detailed account of such tumours. Indeed, there are few references to malignant uterine tumours of any kind (Table I).

TABLE I.—*References to Malignant Tumours of the Uterus of the Mare*

| Author | Year | Number of animals affected | Remarks |
|---|---|---|---|
| *(i) Positive findings* | | | |
| Sticker | 1902 | 8 | Included in a survey of 509 equine " cancers ". |
| Martel (cit. Feldman, 1926) | 1912 | 1 | One of 86 " cancers " found in 20,000 mares examined in Paris abattoir. |
| Kimura | 1917 | 1 | Included in 142 tumours found in 8 years in 77,224 horses in Tokyo. |
| Ball | 1932 | 1 | " Spindle-cell sarcoma " of body of uterus. |
| Tamaschke | 1951–52 | 6 | In a series of 1447 equine tumours (including 546 carcinomas) collected from literature. |
| Jennings* | 1963 | 1 | Malignant tumour, probably leiomyosarcoma, in uterus of 12-year-old mare. |
| *(ii) Negative findings* | | | |
| Lechner | 1958 | 0 | 28 Equine tumours examined in Münich veterinary school, 1952–57. |
| Cotchin | 1960 | 0 | 90 Equine tumours examined at Royal Veterinary College, London, 1950–60. |
| Kronberger | 1961 | 0 | 1016 Equine tumours examined in Dresden/Leipzig veterinary schools, 1917–59. |
| Miller* | 1963 | 0 | Extensive experience at Equine Research Station, Newmarket. |

* Personal communication.

Reports of squamous-cell carcinoma of the external genitalia of mares include three tumours of the vulva (Hennig, 1903 ; McKenny, 1906 ; Lloyd, 1911) and one of the clitoris (Hennig, 1903) : in London, we have recently seen a vulval carcinoma in a 20-year-old mare. Haygard (1956), in Kentucky, stated that cervical tumours were comparatively rare in mares : " In my experience, two cases of malignancy have been found." Tamaschke (1951–52), in a survey of published cases, listed 5 carcinomas of the vagina and 13 of the " vulva and cervix " (details lacking). Kronberger (1961), surveying 1016 tumours examined in the Dresden and Leipzig veterinary schools, listed 17 tumours of the vagina and vulva, including 11 carcinomas. Recently, Miller (1963, personal communication) has commented on the rarity of cervical tumours in mares, a view supported by the extensive clinical experience of vaginoscopy by practising colleagues.

*Donkey and mule*

I have found no record of carcinoma of the uterus in donkeys, and Montpellier, Samso and Catanei (1952) noted particularly that they had found no allusion in veterinary literature to uterine tumours in mules (horse-donkey cross). Migliavacca (1950) reported a leiomyoma of the left uterine horn of a 14-year-old donkey.

*Cow* (Bos taurus)

Although the fairly general opinion is that uterine cancer is rare in cows, there have been quite a few reports of such tumours (Table II), and Monlux, Anderson, Davis and Monlux (1956) have urged the view that in fact the bovine uterus may be the site of an economically-important adenocarcinoma. In their key paper on bovine uterine adenocarcinomas found at meat inspection, these authors reported 26 cases in cows : these animals ranged from 2 to 12 years of age, and all but one of those for which the breed was known were Herefords (although this may be of no significance, in view of the predominance of this breed in the slaughtered animals under examination). It was pointed out that small tumours might be overlooked in abattoirs, where the uterus receives only cursory inspection, and also that few cases on record were from animals showing clinical signs. In these 26 cases, metastases were recognized in the lungs (22 animals), bronchial (12), mediastinal (14), sub-lumbar (15) and internal iliac nodes (17), ovary (5) and peritoneal surfaces (14), but not in the brain, adrenal, kidney, skeletal muscle or bone-marrow.

Most of the tumours originated in the uterine cornua—in a few instances a single tumour involved both horns. The free part of the horn was the chief site. The tumours tended to form sclerotic, annular constrictions, without perforation of the serosa or extension on to the endometrial surface. The tumours were histologically adenocarcinomas with a very fibrous stroma. No primary ovarian tumours were noted in association with these carcinomas.

Reference was made by Monlux and his colleagues to 62 cows which had metastatic lesions indistinguishable from metastatic lesions of known primary uterine carcinomas. They suggested that adenocarcinoma of the bovine uterus might rank only behind squamous-cell carcinoma of the integument of the eye and its appendages, and lymphoid tumours, in causing economic loss to the cattle industry.

These observations are supplemented by those of Brandly and Migaki (1963). In a total of 737 tumours (484 malignant, 253 benign) from cattle, that were included in the first one thousand neoplasms submitted for histological examination to the Biological Sciences Laboratories of the United States Department of Agriculture since 1954, there were included 116 adenocarcinomas of the uterus, making this the second of the five most common cattle tumours submitted—others being malignant lymphoma 177, neurofibroma and neurilemmoma 81, adrenal tumours 61, and " eye cancer " 58. These figures are clearly incomplete, from the way in which the specimens were collected—indeed, Brandly (1963, personal communication) stated that it had been estimated by the veterinary meat inspectors concerned that probably not more than one uterine tumour in a hundred

TABLE II.—*References to Malignant Tumours of the Uterus of the Cow*

| Author | Year | Number of animals affected | Remarks |
|---|---|---|---|
| Guillebeau | 1899 | 7 | Carcinomas, of which 5 were said to be of cervical origin. Five of the 7 had metastasized. |
| Sticker | 1902 | 17 | In 110 " cancers " of cattle (plus 3 vaginal " cancers "). |
| Detroye | 1906 | 5 | In 49 bovine " cancers " at meat inspection, 1890–1902; 4 " epitheliomas ", 1 sarcoma. |
| Trotter | 1906, 1911 | 1 | In 305 primary tumours in 300 abattoir cattle, Glasgow. |
| Wyssmann | 1912 | 1 | Non-metastasizing carcinoma in 7-year-old 280-day pregnant cow. |
| Scholer | 1916 | 2 | Carcinomas in cows 8 and 10 years old; in 12,000 cattle in Basle abattoir; both have ovarian " mestastases ". |
| Cadiot | 1921 | — | " Relatively common " uterine carcinoma. |
| Deme (cit. Montpellier *et al.*, 1952) | 1926 | 9 | Carcinomas. |
| Udall, Fincher and Cushing | 1926 | 1 | Fatal metastasizing carcinoma, involving cervix and uterus, in 6-year-old cow. |
| Karetta | 1928 | 1 | Metastasizing adenocarcinoma. First uterine carcinoma in about 166,000 cattle in 6 years at Vienna abattoir. (Detailed histology). |
| Eyer | 1929 | 2 | Two carcinomas, one with mammary gland(!) metastases. |
| Sedlmeier | 1930 | 1 | Large metastasizing scirrhous carcinoma of corpus, found at meat inspection. |
| Thieulin | 1930 | 1 | Carcinoma in 5-year-old cow. |
| Davis, Leeper and Shelton | 1933 | 4 | Carcinomas of uterus found in 90 bovine tumours in 5 years at meat inspection in Denver. |
| Faure | 1936 | 1 | Metastasizing uterine carcinoma, weighing 45 kg.(!) in 6-year-old cow (also reported by Ball and Boudet, 1925). |
| Verardini | 1937 | 4 | " Myoepitheliomas " (3 with myomatous and carcinomatous components; one with both muscle and epithelium malignant—both metastasizing). |
| Buer | 1942 | 4? | One, possibly 4, of 33 bovine pulmonary tuberculous-like lesions, thought to be of uterine carcinoma origin. |
| Harbitz | 1942 | 3 | Carcinomas. |
| Ottosen | 1943 | 14 | Scirrhous carcinomas; 8 with pulmonary metastases. |
| Lagerlöf and Boyd | 1953 | 1 | Metastasizing uterine carcinoma (found at autopsy of over 6,000 cows). |
| Monlux, Anderson, Davis and Monlux | 1956 | 26 | (See text). |
| Monlux, Anderson and Davis | 1956 | 1 | Metastasizing carcinoma in the uterine horn of pregnant cow, foetus in other horn. |
| Overgoor | 1958 | 2 | Metastasizing uterine carcinomas. |
| Kronberger | 1961 | 5 | Five carcinomas in total of 26 uterine tumours in a series of 738 bovine tumours. |

TABLE II—*continued*

| | | | |
|---|---|---|---|
| Brandly and Migaki | 1963 | 116 | Uterine adenocarcinomas (see text). |
| Campbell* | 1963 | 3 | Three uterine carcinomas—in 16 years (Glasgow). |
| Dow* | 1963 | 1 | In a series of 500 " screw " cows examined post mortem in Glasgow. |
| Head* | 1963 | 4 | In 467 bovine tumours examined in Edinburgh, 1940–57. |
| Jennings* | 1963 | 1 | Uterine adenocarcinoma (Liverpool). |
| Machado* | 1963 | 1 | In a series of 135 bovine tumours in Minas Gerais, Brasil. |
| McEntee* | 1963 | 5 | From Cornell (see text). |
| Misdorp* | 1963 | 6 | From Amsterdam (see text). |
| Moulton* | 1963 | 1? | A possible metastasizing uterine adenocarcinoma (from Davis, California). |
| dos Santos* | 1963 | 2 | Two uterine carcinomas in 189 bovine tumours (from Rio de Janeiro). |
| Smit* | 1963 | 1 | Metastasizing carcinoma (from Onderstepoort). |

* Personal communication.

would have been submitted for microscopic examination. Brandly's series were generally in cows, 5 or more years of age, although one animal was reported to be 2 years of age, and a few were 3 or 4 years old.

McEntee (1963, personal communication) has kindly provided me with details of 5 cases of bovine uterine carcinoma from Cornell—a metastasizing uterine adenocarcinoma in the right horn and body of a 15-year-old Holstein ; a scirrhous carcinoma in the left and right cornua of an aged Holstein, which also showed chronic cervicitis and bilateral cystic ovaries ; a metastasizing uterine adenocarcinoma in a 12-year-old Holstein ; a metastasizing uterine adenocarcinoma, arising at the junction of cervix and body, of a 5-year-old Holstein (also showing cystic endometrial glands and chronic metritis) ; a carcinoma, affecting each cornu, in a 6–7-year-old Ayrshire. From Amsterdam, Misdorp (1963, personal communication) has also sent details of a series of 6 metastasizing uterine adenocarcinomas in cows ; these were found in about 150,000 cows slaughtered in a period of about 3½ years. The sites of the lesions were in the body of the uterus, in the left uterine horn, in the right uterine horn, in both horns, and in the left uterine horn, in five cases respectively.

Some correspondents have reported negative observations. Ajello (1963, personal communication) in Sicily, Barboni (1963, personal communication) in Italy, and Curial (1963, personal communication) in Brazil have no records of cases of uterine (or cervical) carcinoma in cows, nor indeed, in other species. Dow (1963, personal communication), examining over 2000 genitalia from " cast " dairy cows examined in Glasgow, found no uterine tumours although, as shown in Table II, in a series of about 500 " screw " cows he autopsied there was one uterine adenocarcinoma. Jackson (1963, personal communication), with experience in South Africa and elsewhere, has never seen a carcinoma of the uterus (body or cervix) in cows. Ressang (1963, personal communication) states that uterine or cervical carcinoma has never been seen in cows in Indonesia nor, for that matter, in any other species. Trein (1963, personal communication) in Porto Alegro, Brazil, has no examples of uterine tumours in his collection of 500 animal neoplasms. Carcinomas of cervix, vagina or vulva are occasionally reported in cows (e.g. Paine, 1909 ; Trotter, 1911 ; Joest and Biedermann, 1921 ; Ingmire, 1947). Monlux, Anderson and Davis (1956) reported one vulval carcinoma and Monlux, Anderson, Davis and Monlux (1956) 5 carcinomas—2 of cervix, one of vagina and 2 of vulva. Head (1963, personal communication) in Edinburgh, saw one carcinoma of " vagina and cervix ".

Turning to the benign tumours of the female genital tract, Lelièvre (1946), who collected 87 cases of " fibroids " in animals from the literature, found that they were most frequently reported in cows. Leiomyomas of the uterus have been reported by Vandeplassche and Thoonen (1950) (the tumours weighed 280 kg.) and Wargniez (1953). Signol (1951) found a fibromyoma of the cervix, and Cotchin (1960) recorded 20 vaginal tumours—7 fibromas, 7 lipomas, 3 fibromyomas, 2 leiomyomas and 1 fibropapilloma. Kronberger (1961), in 26 uterine tumours, included 1 fibroma and 15 leiomyomas, and in 88 vaginal tumours, 51 fibromas, 23 lipomas and 1 fibropapilloma.

Other miscellaneous tumours have been recorded in the bovine female genital tract : the nature of some of these lesions is obscure. Feger's case (1897) is clearly misdiagnozed as a carcinoma. Soldati (1929) reported a myosarcoma ; Ball (1932)—2 sarcomas ; Ball, Zaessinger and Martin (1933)—" môle hydatiforme embryonnée " ; Karlson and Kelly (1941)—" chorio-hemangioma " ; Malinziewicz and Kramarz (1949)—a " tumour " causing sterility ; Müller (1954)—myeloid leukosis of uterus ; Hewetson and Carter (1955)—neurofibrosarcoma of uterus of a 16-year-old cow ; Mammoli (1959)—2 cases of vaginal " lipophagic granuloma " ; Kronberger (1960)—sarcoma of cervix (one of the only 2 tumours found in 648 autopsied cattle). The nature of the curious uterine lesions reported as occurring on an almost endemic scale by Faure (1936) is uncertain, but the thesis is interesting.

*Sheep* (Ovis aries) *and goat* (Capra hircus)

I have found no record of carcinoma of the uterus or cervix in sheep, but Brandly (1963, personal communication) listed a uterine adenocarcinoma in a goat. Feldman (1931) reported 2 uterine leiomyomas in sheep and Davis, Leeper and Shelton (1933) one leiomyoma and one leiomyosarcoma in sheep. Tamaschke (1951–52) found, in 114 recorded sheep tumours, 5 uterine leiomyomas, one sarcoma and one myosarcoma. Monlux, Anderson and Davis (1956) noted a metastasizing squamous-cell carcinoma of the ovine vulva. Kronberger (1961), in 24 caprine tumours, listed 1 fibroma of the uterus, 1 adenoma of the vagina and vulva and 3 fibromas of the vagina and vulva. Barboni (1963, personal communication) reported a mucous cystadenocarcinoma of the cervix of a goat.

*Sow* (Sus scrofa domestica)

Tamaschke (1951–52) listed 29 female genital tumours in 188 reported tumours of pigs. Nine were uterine—7 leiomyomas, 1 myosarcoma and 1 carcinoma ; this latter tumour is possibly that of Loeb (1900, cited by Slye, Holmes and Wells, 1924). Boucek (1906), in Prague, referred to a sub-serous nodular fibromyoma in a sow. Genest and Trepanier (1952) found a uterine leiomyoma, weighing 118 lb., in a sow, and Gimbo (1955) an endometrial polyp. Monlux, Anderson and Davis (1956) classified a vaginal lesion of a 6-month-old pig as an " embryonic sarcoma ".

Kronberger (1961), in 99 submitted tumours, listed 6 of the uterus (1 sarcoma, 5 leiomyoma).

*Bitch* (Canis familiaris)

I have found no good published photomicrographs of canine uterine carcinoma. There are a few reported cases (Table III), some of doubtful significance. The

TABLE III.—*References to Malignant Tumours of the Uterus of the Bitch*

| Author | Year | Number of animals affected | Remarks |
|---|---|---|---|
| Casper | 1899 | 1 | In 51 " cancers " of dogs. |
| Sticker | 1902 | 4 | In 956 " cancers " of dogs. |
| Cadiot | 1921 | 2 | In 760 " cancers " of dogs. |
| Ball | 1932 | 1 | " Encephaloid carcinoma " of cervix. |
| Schlotthauer | 1939 | 1 | Carcinoma of left cornu of 8-year-old bitch. |
| Tamaschke | 1951–52 | 4 | In 773 carcinomas of dogs (112 of female genitalia). |
| Montpellier, Samso and Catanei | 1952 | 1 | " Epithelioma " of uterine body. |
| Krook | 1954 | 5 | In 301 bitches with carcinoma, autopsied in Stockholm Royal Veterinary College. |
| Lechner | 1958 | 3 | 2 Carcinoma, one adenocarcinoma, in 709 canine tumours (Münich, 1952–57). |
| Kronberger | 1961 | 1 | Uterine carcinoma in 2002 tumours. |
| Andersen | 1963 | 1 | Metastasizing carcinoma, in 10-year-old Beagle, received 300 r (4 × 75 r, 7-day intervals) at 11 months of age. |
| Moulton* | 1963 | 1? | Possible uterine carcinoma (Davis, California). |
| Smith* | 1963 | 1 | Corpus carcinoma (Guelph). |

* Personal communication.

writer (Cotchin, 1959), in a total of 4187 tumours of dogs examined in London, found no uterine or cervical carcinomas, and none have been seen since in some 2000 further canine tumours. Dow (1963, personal communication) in a careful post-mortem study of over 500 bitches, also found no uterine or cervical carcinomas. Head (1963, personal communication) in a series of 8093 tumours from dogs, recorded no uterine carcinomas, but his series included 3 carcinomas of " cervix and vagina ". Vaginal carcinomas in bitches have also been reported by Sticker (1902), Boucek (1906) and Kronberger (1961).

Fibrous and muscular tumours of the female genital tract are common in the bitch, occurring chiefly in the vagina. Auger (1910) reported a cystic fibromyxoma of the cervix. Segolini (1930) referred to myomas of the uterus. Lechner (1958) listed 14 vaginal fibromas. Kronberger (1960), in 743 tumours from autopsy cases, found records of 4 fibromas and 3 leiomyomas of the uterus and, in submitted specimens (Kronberger, 1961), 12 leiomyomas of the uterus and 61 fibromas and 21 leiomyomas of the vagina. Thrasher (1961) reported a uterine leiomyoma. Dow (1963, personal communication) has seen a leiomyosarcoma of the uterine body in a bitch, with regional node deposits. Head's (1963, personal communication) series included 10 cases of adenomyosis of the uterus.

Lesions diagnosed as chorionepitheliomas have been reported occasionally in the bitch (Schlotthauer, 1939 ; Riser, 1940, 1942), but they do not correspond to the human condition.

*Cat* (Felis catus)

Uterine carcinoma has been reported in the cat by Teutschlaender (1920) (4 cases are mentioned without details) ; Boucek (1906) described a squamous-cell carcinoma of the cervix, containing numerous giant-cells ; Ladkany (1943) ; Tamaschke (1951–52) ; Montpellier *et al.* (1952)—in Algiers, " epithelioma " of the cervix. Meier (1956) referred to a cervical squamous-cell carcinoma reported by Collignon (1936) and a uterine " endothelioma " reported by Ball (1932), and described 2 cases of his own—a carcinoma in an 11-year-old spayed cat, in which a portion of the uterus had remained, and a squamous-cell carcinoma in a 5-year-old cat—perhaps derived from metaplastic epithelium, the result of a long-standing pyometra. Lechner (1958), in 32 tumours of cats, included one uterine sarcoma.

In London, we have seen one case of uterine carcinoma in a cat : the affected animal, an 11-year-old nulliparous Persian, had for 3–4 months been showing a serous vaginal discharge. At operation, the uterine cornua were enlarged—one measured 10 by 3 cm., the other $8\frac{1}{2} \times 1\frac{1}{2}$ cm. The walls of the cornua were thin, and they were filled with a serous fluid. At about the mid-point of the larger cornu, there was a depression, about 1 cm. wide and deep, with walls 0·5 cm. deep. Section showed a primary adenocarcinoma. The subsequent history of the cat is unknown.

Dow (1963, personal communication), in almost 250 cats examined in 6 years, found an anaplastic adenocarcinoma of one horn in a cat, with metastases in the sublumbar and para-aortic nodes and lungs. Head (1963, personal communication) saw no uterine or cervical carcinomas in 413 tumours of cats, but noted 6 cases of adenomyosis uteri. Howell (1963, personal communication), from Liverpool, reports a metastasizing adenocarcinoma of the uterus of a 6-year-old cat.

*Rabbit*

There are quite a few reports of endometrial tumours in rabbits (Table IV). It is not clear in all cases whether the tumours were adenomas or adenocarcinomas —indeed, both forms may occur even in the same uterus.

Particular interest attaches to the work of Greene and his colleagues. In considering their reports, it should be remembered that they were dealing with a particular rabbit colony (for composition and management, see Greene, 1935), the circumstances of which may have been peculiarly favourable for the development and observation of uterine tumours.

Greene and Saxton (1938) found 83 rabbits with uterine adenomas or adenocarcinomas in a colony of approximately 500 does in 4 years. These cases followed a move of the Rockefeller Institute rabbitry from New York to Princeton (Greene, 1937). Eight rabbits died from metastasis. The average age at discovery of the tumours was 45 months. The tumours were confined to multiparous does, but disturbances of reproductive function always preceded detection of tumours. The early lesions were pedunculated growths, or small thickenings, of the endometrium, usually on the endometrial folds, adjacent to the endometrial insertion.

Changes present in the adrenal, thyroid, pituitary and mammary glands (including mammary cysts and tumours) were similar to those seen in oestrone-treated mice. Successful auto-, homo-, and hetero-transplantations (guinea-pig eye) were reported.

Greene (1939*a*) discussed transplantation trials ; transplanted tumours were unaccompanied by the pituitary, thyroid and adrenal changes associated with primary tumours. Greene (1941), noting the similar breed susceptibility to uterine tumours and to what he called " toxaemia of pregnancy " (Greene, 1935,

TABLE IV.—*References to Endometrial Epithelial Tumours of the Rabbit*

| Author | Year | Number of animals affected | Remarks |
|---|---|---|---|
| Lack | 1900 | 1 | Malignant tumour. |
| Shattock | 1900 | 1 | Uterine carcinoma. |
| Wagner | 1905 | 1 | Multiple tumours. |
| Selinow | 1908 | 1 | Carcinoma in a rabbit, dying 80 months after unilateral nephrectomy. |
| Boycott | 1911 | 4 | One in pregnant doe. Origin at sites of implantation suggested—? subsequent to abortion. |
| Leitch | 1911 | 1 | Three tumours in a 2½-year-old rabbit (see text). |
| Marie and Aubertin | 1911 | 1 | Cancer of uterus of 9-year-old rabbit. |
| Katase (cit. Polson, 1927) | 1912 | 1 | |
| Stilling and Beitzke | 1913 | 13 | 25 Tumours (adenomas and adenocarcinomas) in 13 animals. |
| Dible | 1921 | 1 | A demonstration case. |
| Koyama | 1927 | 1 | Five separate adenomas (active mammary glands). |
| Polson | 1927 | 4 | Carcinomas (accompanied by abnormal endometrium, plus one case of metastasizing uterine myosarcoma). |
| Rusk and Epstein | 1927 | 1 | Metastasizing carcinoma, in 4½-year-old virgin doe. |
| Usawa | 1930 | 1 | Eleven adenocarcinomas in uterus of 5-year-old multiparous doe. |
| Watrin and Florentin | 1930 | 1 | 3-year-old doe, lactating, but not pregnant for 2 years : tumour said to be of decidual origin. |
| Fardeau | 1931 | 2 | Literature reviewed (rabbit uterine tumours). |
| Cutler | 1934 | 1 | Literature reviewed (rabbit tumours in general). |
| Twort | 1937 | 1 | Carcinoma in 7-year-old doe (splenic metastases). |
| Greene and Saxton | 1938 | 83 | (See text). |
| Orr and Polson | 1938 | 5 | 4 Adenocarcinomas, and one possible sarcoma. Two of the rabbits, although segregated for 1½ years and 4 years respectively, had " distinct mammary activity ". |
| Witherspoon | 1938 | 1 | No metastases. |
| Burrows | 1940 | 15 | (See text). |
| Lombard | 1959 | 3 | Found in animals aged about 2 years in a commercial colony of some 500 white Angora rabbits. |
| Head* | 1963 | 2 | Adenocarcinomas. |

* Personal communication.

1937, 1938, 1939*b*), suggested that the failure of the liver to inactivate oestrogen might be the mechanism involved in tumour formation. Greene and Newton (1948) described the developmental stages of uterine tumours in biopsies and in transplants. Greene and Strauss (1949) recorded that, in a colony of rabbits in the 17-year period ending in 1948, 1100 females came to autopsy. Of these, 234 had tumours, in 55 instances multiple primary tumours. In all but two animals, one of the multiple tumours was an adenocarcinoma of the uterine fundus. Mammary adenocarcinomas were present in 21 of the 55. In 5 instances the uterine carcinomas were accompanied by uterine myomas, and in 4 by uterine myosarcomas.

Burrows (1940 ; see also Burrows and Boyland, 1938) found uterine tumours in 15 of 25 non-inbred rabbits dying at 900 days or later ; 11 of the 15 had primary growths in both cornua. Eleven tumours had metastasized. Progestational changes might be present next to the tumours. Cystic mastopathy was present in 14 of the rabbits and, in 3 instances, there were multicentric mammary tumours, probably malignant.

Tamaschke (1951–52, 1955) listed recorded tumours in rabbits, but overlooked Greene's many cases. In London, we have seen a case of multiple uterine adenomas in a doe, but enquiry of a number of establishments, commercial and scientific, dealing with a large number of rabbits, has not elicited any current cases of uterine tumours in rabbits, although it may be that the animals concerned were too young.

Turning to the cervix, and in contrast to the uterus, Greene (1941) commented that no instance of cervical cancer had been seen by him in 849 rabbits, two or more years old. Greene, Newton and Fisk (1947) found 3 cases of vaginal carcinoma associated with uterine carcinoma. The absence of a squamous-columnar junction in the rabbit cervix, and its presence lower in the vagina, suggested a reason for the absence of cervical cancer in rabbits, and for the position of the three vaginal tumours.

*Mouse*

Spontaneous carcinomas and sarcomas of the mouse uterus have been reported by a few authors (Table V). Andervont and Dunn (1962), incidentally, did not record any uterine tumours in wild house mice kept in captivity.

*Rat*

Although no uterine tumours were reported by McCoy (1909) in about 100,000 rats examined at the Federal Plague Laboratory in San Francisco in 1908–9, and Bullock and Rohdenburg (1917) also reported negative findings in laboratory rats, there are some reports of uterine malignancies (Table VI). For example, Bullock and Curtis (1930) found 48 uterine tumours in 521 primary tumours of 489 rats, including non-cervical squamous-cell carcinoma (10—all but one formed diffuse or nodular thickening of the walls of uterine abscesses), adenocarcinoma (4), adeno-acanthoma (1), carcinoma (1), carcino-sarcoma (4) and sarcoma (23—2 myosarcoma, 2 fibrosarcoma, 2 pleomorphic-cell, 1 spindle-cell and 16 mixed-cell sarcoma).

Hanau (1889), in his classic paper from Zürich, reported a spontaneous metastasizing transplantable " cancroid " of the vulva. Singer (1913–14) passed a

TABLE V.—*References to Malignant Tumours of the Uterus of the Mouse*

| Author | Year | Number of animals affected | Remarks |
|---|---|---|---|
| Woglom | 1919 | 1? | Tumour of uncertain type, with hepatic metastasis, in 22-month-old mouse. |
| Slye, Holmes and Wells | 1924 | 7 | Seven sarcomas (plus 11 leiomyomas, 3 adenomas, 1 teratoma) in 39,000 autopsied mice. |
| Heidenhain | 1929 | 1 | Two tumours in one uterus (doubtful carcinomas). |
| Strong | 1938 | 3 | Sarcomas in 3 CBA mice. |
| Snell | 1956 | 2+ | Carcinomas in 2 hybrid mice (adenomas in 2 DBA mice) and a number of sarcomas. |
| Gardner and Pan | 1948 | 13 | 13 of 56 Female mice of PM strain had uterine or vaginal tumours—5 squamous-cell carcinomas, 7 undifferentiated epithelial tumours, 1 spindle-cell sarcoma. |
| Guérin | 1954 | 3 | Three sarcomas (plus one fibromyoma). |
| Fischer and Kühl | 1958 | 2 | Corpus cancers in 2 of 1572 female mice. |
| Dunn* | 1964 | — | A number of adenocarcinomas in old BALB/c mice (many have been successfully transplanted). |

* Personal communication.

TABLE VI.—*References to Malignant Tumours of the Uterus of the Rat*

| Author | Year | Number of animals affected | Remarks |
|---|---|---|---|
| Bullock and Curtis | 1930 | 48 | In 521 primary tumours of 489 rats (see text). |
| Curtis, Bullock and Dunning | 1931 | — | 0·6 per cent incidence of uterine tumours in female rats over 13 months of age. |
| Guérin and Guérin | 1934 | 1 | Adenocarcinoma (transplantable). |
| Ratcliffe | 1940 | 7 | Five malignant epithelial tumours and 2 sarcomas in rats of Wistar Institute. |
| Guérin | 1954 | 18 | 2 Sarcomas, 13 carcinomas, 3 mixed tumours (plus 27 benign tumours). |
| Crain | 1958 | 3 | Metastasizing adenocarcinomas (in 200 tumours of 189 tumour-bearing albino rats 18–24 months old). |
| Gilbert and Gilman | 1958 | 12+ | 11 Adenocarcinomas and 1 myosarcoma (plus 1 carcinoma *in situ*, and 2 vaginal carcinomas) in 377 tumour-bearing female rats. |
| Schulze | 1960 | 23 | Carcinomas, in 34 spontaneous tumours of uterus and ovary amongst 66 tumours in 1373 autopsied Sprague-Dawley and Bethesda Black rats. |
| Thompson *et al.* | 1961 | 2 | Leiomyosarcomas (in 2 of 82 Sprague-Dawley rats). |
| Snell* | 1964 | 7 | Tumours of uterine horns: 1 adenocarcinoma, 1 squamous-cell carcinoma, 1 sarcoma, in 23 Buffalo rats; 1 angiosarcoma in 20 M520 rats and 2 in 23 F344 (Fischer) rats; 1 adenocarcinoma in 29 AC I (A × C) rats. (All rats over 18 months old.) |

* Personal communication.

uterine sarcoma successfully through 7 generations, and Pollia (1938) found a uterine sarcoma which was 100 per cent transplantable.

*Guinea-pig*

I have found no record of uterine or cervical cancer in the guinea-pig (endometrial hyperplasia was reported by Geil and Davis, 1962).

*Hamster* (Cricetus auratus)

Fortner (1957) found an endometrial carcinoma in 1 of 142 females. Rivière, Chouroulinkov and Guérin (1960) found uterine carcinoma in 2 of 40 irradiated female hamsters—these had received 300 r of general irradiation.

*Other miscellaneous species*

A number of spontaneous uterine tumours have been reported sporadically in other species (Table VII). In passing, attention may be drawn to the invaluable review of diseases of wild animals and birds by Halloran (1955).

TABLE VII.—*References to Uterine Neoplasms in Miscellaneous Species of Wild and Zoo Animals*

| Author | Year | Remarks |
|---|---|---|
| Murray | 1908 | Squamous-cell cervical carcinoma in a gazelle (Petit's case). |
| Betke | 1911 | " Non-metastasizing papillary adenocarcinoma " over portio cervix of 40-year-old Indian rhinoceros (Frankfurt Zoo) (plus myxofibroma of vagina and uterus). |
| Fox | 1912 | Uterine adenocarcinoma in 3 wild swine (*Sus scrofa*). |
| Schurmann | 1915 | Uterine fibroma in elephant, leiomyoma in black lemur, " chorionepithelioma " in Canada porcupine. |
| Boyd | 1929 | Fibroma of uterus in axis deer. |
| Ratcliffe | 1932 | Endometrial cystadenoma in yellow baboon. |
| Graves | 1937 | Uterine adenocarcinoma in a mink. |
| Rewell and Willis | 1949 | Uterine fibromyoma (4 cm. long) in a whale. |
| Halloran | 1955 | Carcinoma of cervix of monkey (p. 72); adenocarcinoma of uterus of coypu (p. 137), in lions (p. 214), in antelopes (p. 299). |
| Hisaw and Hisaw | 1959 | Metastasizing cervical carcinoma in monkey (*Macaca mulatta*). |
| Lombard and Witte (see also Ratcliffe, 1933) | 1959 | At Philadelphia Zoo: Uterine carcinoma in silver-blue mink, a gemsbok, a sable antelope, 2 European wild boars, a coypu, and a Branick's pacarana (*Dinomys branichii*). |
| Newberne and Robinson | 1960 | Undifferentiated carcinoma, uncertain origin, involving left ovary, oviduct and anterior aspect of uterus, of *Macaca philippinensis*. |
| Sternberg | 1961 | Cervical carcinoma *in situ* in *Macaca mulatta*. |
| Kronberger | 1962 | Leiomyoma of uterus in long-tailed monkey (Meerkatze) and fibroma of uterus in 28-year-old *Elephas maximus*. |

### DISCUSSION

Much more work needs to be done before comparative pathology can provide information and interpretations that will help to clarify thinking about human uterine cancer. It may well be that future study will merely confirm the present somewhat negative findings, but even negative findings are in this sense significant: it is clear that, should it be established that uterine cancer, particularly in its cervical form, is mostly a human disease, this fact alone cannot but be of central importance in any consideration of possible aetiological factors.

While available information is admittedly fragmentary, the tentative conclusion can in fact be drawn from this present survey that cervical carcinoma is for all practical purposes a disease of women only, but that corpus carcinoma is predominantly but rather less restrictedly a human disease—it occurs in many species of animals, although rarely, while in two species—rabbit and cow—it may be less rare, although even for these two animals we need information about its present incidence. Jubb and Kennedy (1963) go so far as to state baldly, in their magisterial textbook on the pathology of domestic animals, in a chapter prepared in collaboration with McEntee, that carcinoma of the endometrium and cervix are rare neoplasms in domestic animals, and that this rarity is real, and not merely apparent because of inadequate post-mortem examination. Leaving aside the discrepancy between this view, which must carry much weight, and that advanced by Monlux, Anderson, Davis and Monlux (1956) on the possible importance of bovine uterine carcinoma, we should always remember the many difficulties that prevent us from forming anything like as clear a picture of the incidence of tumours in animals as we can in humans. There is, for example, no compulsory registration of the cause of death. In the case of food animals, abattoir examination of the female genitalia is generally only cursory, and in any event abattoir findings as regards neoplasia are generally very inadequately recorded, confirmed and reported. Another important consideration is that domestic animals (other than household pets) rarely live out their potential life span, and cows will in any case not be likely to be kept if their breeding capacity is interfered with. However, as we have seen, even in those animals (horse, dog, cat) which do tend to live to old age, uterine tumours are rarely reported.

The work of Monlux and his colleagues is of great interest, and it would seem to be of particular importance that their opinion as to the economically-significant incidence of bovine uterine carcinoma should be confirmed by further intensive studies on cattle populations under varying management systems in different countries. Particular attention needs to be paid to two points raised by these workers. First, the origin of bovine endometrial carcinoma should be studied in early cases—they found lesions to lie deep in the endometrium. Second, they jound no significant endometrial or ovarian abnormalities to be related to these tumours.

Respecting this second point, Jubb and Kennedy (1963) state that the most popular current opinion on the pathogenesis of endometrial carcinomas in women relates them to prolonged oestrogenism, cystic hyperplasia of the same cause being the pre-cancerous lesion. They point out that while cystic endometrial hyperplasia is common in animals, uterine carcinoma is generally rare, and they further point out that when it does occur, it does not seem to require oestrogenic conditioning of the endometrium.

Cystic endometrial hyperplasia in mares is rather rare ; a single case has been studied intensively by Zebracki (1962). Granulosa-cell and other ovarian tumours do occasionally occur in mares, but the cause of cystic endometrial hyperplasia in these animals is not known (Jubb and Kennedy, 1963). In cattle, Jubb and Kennedy (1963) state that cystic endometrial hyperplasia is invariably associated with ovarian follicular cysts, or with granulosa-cell tumours. In sheep (Fedrigo, 1950 ; Barrett, Moule, Braden and Harris, 1961), on the other hand, the significant cause of cystic endometrial hyperplasia is exogeneous, being due to oestrogenic substance in pasture legumes (chiefly subterranean or red clover). Endometrial hyperplasia occurs by no means rarely in cats (Guttmacher, 1924 ; Dow, 1962), but it is of commonest occurrence in the bitch, where, although in some cases it is associated with apparently oestrogenic ovarian tumours (often granulosa-cell tumours, Cotchin, 1961), in most cases it seems to develop in the middle-aged bitch which has bred infrequently if at all, apparently occurring as a result of hormonal disturbances. The work of Dow (1957, 1958, 1959*a* and 1959*b*) in fact indicates that the pyometra complex in the bitch can be reproduced by cyclic treatments with oestrogen and progesterone, the inflammatory reaction supervening on endometrial hyperplasia being progesterone-dependent. In this connection, too, reference should be made to the leiomyoma (clinical " fibroid ") which, according to Jubb and Kennedy (1963), is the commonest tumour of the tubular genitalia of the bitch (a statement with which we would agree if the term includes fibrous lesions of the vagina). The bitch " fibroid " is said to be hormone dependent, being probably always associated with ovarian follicular cysts or with oestrogen-secreting ovarian tumours, and often also accompanied by endometrial hyperplasia and by mammary glandular hyperplasia or neoplasia. Jubb and Kennedy state that bitches castrated early in life do not develop " fibroids ", and that established tumours regress following castration.

Now, in attempting a fruitful comparison of animals with women, we have of course to bear in mind many differences, e.g., in their oestrus cycles and in the associated hormonal conditions, in the nature of the cyclic endometrial changes, the presence or absence of a menopause and so on, that may lead to confusion, but perhaps the animal in which a situation occurs that is most analagous to that of the woman who is prone to develop uterine corpus carcinoma is the bitch, of low or nil parity, in middle life. The bitch, however, although it commonly develops endometrial hyperplasia, apparently rarely develops uterine endometrial carcinoma. How can this be accounted for? It may be suggested that one of the significant differences between woman and bitch might be found in the relatively prolonged progesterone phase that occurs in the oestrus cycle of the latter. Perhaps the bitch of low or nil parity is protected from developing uterine cancer because it has few oestrus cycles (two per year), and because in these it always goes through a prolonged progesterone stage—if not of pregnancy, then invariably of pseudo-pregnancy.

Again, perhaps the bitch is more prone to develop pyometra as a sequel to endometrial hyperplasia, often apparently from superimposed bacterial infection of the progesterone-sensitized uterus, and then either dies, or is subjected to curative hysterectomy, before it has time to develop endometrial cancer.

It would seem that the work of Pierson (1935, 1937*a*, *b*, 1938*a*, *b*) and of Meissner, Sommers and Sherman (1957) and Sommers and Meissner (1957) on the experimental production of endometrial hyperplasia and neoplasia in rabbits by oestrogenic substances might be worth repeating, and extending to test the possible significance of progesterone. At the same time, further study is needed of spontaneous uterine tumours in rabbits, domestic or laboratory. The rabbit tumours differ somewhat from human corpus carcinomas, but they appear to be related to endometrial hyperplasia, and to have a hormonal origin.

As regards cervical cancer, although this has been produced experimentally in laboratory animals, it does not appear to be a significant spontaneous disease in any species of animal, large or small. This might suggest that cervical cancer is due to specifically human factors, including possible peculiarities of structure (the absence of a squamous-columnar junction from the rabbit cervix has already been mentioned), and hormonal relationships which are at present unclarified. So-called cervical " erosions ", for example, seem to be a purely human condition, although Miller (1963, personal communication) draws attention to the occurrence in the cervix of the mare of lesions due to tearing at foaling. Again, carcinoma *in situ* has not been reported, so far as I know, in any species other than man and monkey. The aetiological role of smegma has also been widely canvassed. It may be of some relevance that while penile carcinoma is not a very rare tumour of horses, and while horse smegma has been shown to be carcinogenic to the skin of mice (Plaut and Kohn-Speyer, 1947), cervical cancer is not a significant disease of mares. Here the obvious differences between sexual and breeding practices of man and horse may clearly be significant. Incidentally, the carcinogenic fraction of horse smegma should be identified, and it would be of interest to know the species distribution of the smegma bacillus, which an old report (Pellegrino, 1906, cited by Wilson and Miles, 1955) said occurred in the dog, as well as in man and woman.

In conclusion, it seems desirable, firstly, that a more extensive survey of the occurrence and, equally, of the non-occurrence of uterine cancer in animals, domesticated or wild, should be made. There are large areas of the world about which our information is scanty or non-existent, and in this connection Latin America would seem to offer an exciting field of study, with particular reference to its indigenous mammals. Secondly, the work of Monlux and colleagues in respect of the importance of uterine carcinoma in cows presents a challenge that should be accepted, and should lead to further intensive study of cattle in U.S.A. and elsewhere. Thirdly, the hormonal induction and control of uterine cancer should be more extensively studied in the rabbit, an animal in which the spontaneous disease is thought not to be rare, and in which uterine carcinoma has already been produced experimentally.

### SUMMARY

A survey has been made of the available published work on spontaneous uterine tumours in animals—domestic, laboratory and wild—and enquiries have been addressed to veterinary pathologists in various parts of the world. The information so gained is presented in summary form in a series of tables, giving references to uterine tumours in the mare, cow, dog, rabbit, mouse, rat and in wild animals. No animal species is yet known to have a significant incidence of spontaneous carcinoma of the uterine cervix, an observation which, if confirmed, would suggest that cervical cancer in women is due to specifically human factors. Endometrial carcinoma is also predominantly, but less restrictedly, a human disease ; it seems to be uncommon in most species, but it has been reported not rarely in rabbits, and some observations indicate that it may be a tumour of some importance in cows. This otherwise general rarity of endometrial carcinoma is in contrast to the quite common occurrence of endometrial hyperplasia. For example, in the bitch, a species in which endometrial hyperplasia is common, endometrial carcinoma is practically unknown. Three subjects for further study are suggested : First, an extensive survey of the incidence of uterine cancer in animals throughout the world ; second, a study of the incidence of uterine endometrial carcinoma in cows ; third, a study of the experimental induction of uterine cancer in the rabbit, a species in which the spontaneous disease is thought not to be rare, and in which it has already been produced experimentally.

### REFERENCES

ANDERSEN, A. C.—(1963) *J. Amer. vet. med. Ass.*, **143**, 500.

ANDERVONT, H. B. AND DUNN, T. B.—(1962) *J. nat. Cancer Inst.*, **28**, 1153.

AUGER, M. L.—(1910) *J. Méd. vét.*, **61**, 449.

BALL, V.—(1932) *Rev. vét., Toulouse*, **84**, 72.

*Idem* AND BOUDET, P.—(1925) *Ibid.*, **77**, 283.

*Idem*, ZAESSINGER, J. AND MARTIN, P.—(1933) *Bull. Soc. Sci. vét Lyon*, No. 6, p. 333.

BARRETT, J. F., MOULE, G. R., BRADEN, A. W. H. AND HARRIS, A. N. A.—(1961) *Aust. vet. J.*, **37**, 14.

BETKE, R.—(1911) *Frankfurt. Z. Path.*, **6**, 19.

BOUCEK, Z.—(1906) *Arch. wiss. prakt. Tierheilk.*, **32**, 585.

BOYCOTT, A. E.—(1911) *Proc. R. Soc. Med.*, **4**, 225.

BOYD, A. G.—(1929) *Cornell Vet.*, **19**, 33.

BRANDLY, P. J. AND MIGAKI, G.—(1963) *Ann. N.Y. Acad. Sci.*, **108**, 872.

BUER, A. W.—(1942) *Norsk VetTidsskr.*, **54**, 488.

BULLOCK, F. D. AND CURTIS, M. R.—(1930) *J. Cancer Res.*, **14**, 1.

*Idem* AND ROHDENBURG, G. L.—(1917) *Ibid.*, **2**, 39.

BURROWS, H.—(1940) *J. Path. Bact.*, **51**, 385.

*Idem* AND BOYLAND, A. E.—(1938) *Amer. J. Cancer*, **32**, 367.

CADIOT, M.—(1921) *Rec. Méd. vét.*, **97**, 245.

CASPER, M.—(1899) Abstract in *Rev. vét. Toulouse*, **24**, 440.

COLLIGNON, H.—(1936) ‘ Le cancer de l’utérus chez les femelles domestiques. Etude anatome-clinique.’ Thesis, Toulouse.

COTCHIN, E.—(1959) *Vet. Rec.*, **71**, 1040.—(1960) *Ibid.*, **72**, 816.—(1961) *Res. vet. Sci.*, **2**, 353.—(1962) *Bull. World Hlth Org.*, **26**, 633.

CRAIN, R. C.—(1958) *Amer. J. Path.*, **34**, 311.

CURTIS, M. R., BULLOCK, F. D. AND DUNNING, W. F.—(1931) *Amer. J. Cancer*, **15**, 67.

CUTLER, O. I.—(1934) *Ibid.*, **21**, 600.

DAVIS, C. L., LEEPER, R. B. AND SHELTON, J. E.—(1933) *J. Amer. vet. med. Ass.*, **83**, 229.

DETROYE—(1906) *Bull. Soc. cent. Méd. vét.*, **60**, 390.

DIBLE, J.—(1921) *J. Path. Bact.*, **24**, 355.

DOW, C.—(1957) *Vet. Rec.*, **69**, 1409, 1415.—(1958) *Ibid.*, **70**, 1102.—(1959*a*) *J. comp. Path.*, **69**, 237.—(1959*b*) *J. Path. Bact.*, **78**, 267.—(1962) *Vet. Rec.*, **74**, 141.

EYER, P. G.—(1929) ‘ Etude sur les tumeurs du vagin et de l’utérus chez la vache.’ Thesis, Paris (Alfort).

FARDEAU, G.—(1931) ‘ Les tumeurs spontanées chez le lapin. Revue critique.’ Thesis, Paris.

FAURE, C.—(1936) ‘ Contribution a l’étude du cancer utérin chez la vache.’ Thesis, Toulouse.

FEDRIGO, G.—(1950) *Nuova Vet.*, **26**, 232.

FEGER—(1897) *Z. Fleisch- u. Milchhyg.*, **7**, 29.

FELDMAN, W. H.—(1926) *Amer. J. Path.*, **2**, 545.—(1931) *Amer. J. Cancer*, **15**, 2044.

FISCHER, W. AND KÜHL, I.—(1958) ‘ Geschwülste der Laboratoriumsnagetiere.’ Dresden and Leipzig (Theodor Steinkoff), pp. 74–89.

FORTNER, J. G.—(1957) *Cancer*, **10**, 1153.

FOX, H.—(1912) *J. Path. Bact.*, **17**, 217.

GARDNER, W. U. AND PAN, S. C.—(1948) *Cancer Res.*, **8**, 241.

GEIL, R. G. AND DAVIS, C. L.—(1962) *Amer. J. vet. Res.*, **23**, 362.
GENEST, P. AND TREPANIER, M.—(1952) *Canad. J. comp. Med.*, **16**, 271.
GILBERT, C. AND GILMAN, J.—(1958) *S. Afr. J. med. Sci.*, **23**, 257.
GIMBO, A.—(1955) *Nuova Vet.*, **31**, 16.
GRAVES, E. F.—(1937) *J. Amer. vet. med. Ass.*, **91**, 97.
GREENE, H. S. N.—(1935) *J. exp. Med.*, **62**, 305.—(1937) *Ibid.*, **65**, 809.—(1938) *Ibid.*, **67**, 369.—(1939*a*) *Ibid.*, **69**, 447.—(1939*b*) *Proc. Soc. exp. Biol. N.Y.*, **40**, 606.—(1941) *J. exp. Med.*, **73**, 273.
*Idem* AND NEWTON, B. L.—(1948) *Cancer*, **1**, 82.
*Idem*, NEWTON, B. L. AND FISK, A. A.—(1947) *Cancer Res.*, **7**, 502.
*Idem* AND SAXTON, J. A., Jr.—(1938) *J. exp. Med.*, **67**, 691.
*Idem* AND STRAUSS, J. S.—(1949) *Cancer*, **2**, 673.
GUÉRIN, M.—(1954) 'Tumeurs spontanées des animaux de laboratoire (souris-rat-poule).' Paris (Amédée Legrand).
*Idem* AND GUÉRIN, P.—(1934) *Bull. Ass. franç. Cancer*, **23**, 632.
GUILLEBEAU, A.—(1899) *Schweiz. ArchTierheilk.*, **41**, 253.
GUTTMACHER, A. F.—(1924) *Johns Hopk. Hosp. Bull.*, **35**, 49.
HALLORAN, P. O'C.—(1955) *Amer. J. vet. Res.*, **16**, 1.
HANAU—(1889) *Arch. klin. Chir.*, **39**, 678.
HARBITZ, F.—(1942) *Norsk. VetTidsskr.*, **54**, 193.
HAYGARD, C. E.—(1956) *Cornell Vet.*, **46**, 327.
HEIDENHAIN, L.—(1929) *Z. Krebsforsch.*, **28**, 174.
HENNIG—(1903) *Arch. wiss. prakt. Tierheilk.*, **29**, 158.
HEWETSON, R. W. AND CARTER, P. D.—(1955) *Aust. vet. J.*, **31**, 80.
HISAW, F. L. AND HISAW, F. L., Jr.—(1959) *Cancer*, **11**, 810.
INGMIRE, C. W.—(1947) *Vet. Med.*, **42**, 427.
JOEST, E. AND BIEDERMANN, R.—(1921) *Z. Krebsforsch.*, **18**, 51.
JUBB, K. V. AND KENNEDY, P. C.—(1963) 'Pathology of Domestic Animals.' New York and London (Academic Press) Vol. 1. pp. 443 ff.
KARETTA, F.—(1928) *Wien. tierärztl. Mschr.*, **15**, 625.
KARLSON, A. G. AND KELLY, M. D.—(1941) *J. Amer. vet. med. Ass.*, **99**, 133.
KIMURA, T.—(1917) *Gann.*, **11**, 38.
KOYAMA, M.—(1927) *Ibid.*, **21**, 7.
KRONBERGER, H.—(1960) *Mh. VetMed.*, **15**, 730.—(1961) *Ibid.*, **16**, 296.—(1962) *Nord. VetMed.*, **14**, Suppl. 1, 297.
KROOK, L.—(1954) *Acta path. microbiol. scand.*, **35**, 407.
LACK, H. L.—(1900) *J. Path. Bact.*, **6**, 154.
LADKANY, F.—(1943) 'Le cancer chez le chat.' Thesis, Toulouse.
LAGERLÖF, N. AND BOYD, H.—(1953) *Cornell Vet.*, **43**, 64.
LECHNER, M.—(1958) 'Spontantumoren bei Säugetieren. Ein Beitrag zur vergleichenden Geschwulstforschung.' Thesis, Münich.
LEITCH, A.—(1911) *Proc. R. Soc. Med.*, **5**, 1.
LELIÈVRE, A. Y.—(1946) 'Les corps fibreux de l'utérus en pathologie comparée.' Thesis, Paris (Alfort).
LLOYD, L. W. W.—(1911) *Vet. J.*, **67**, 634.
LOMBARD, C.—(1959) *Bull. Acad. vét. Fr.*, **32**, 447.
LOMBARD, L. S. AND WITTE, E. S.—(1959) *Cancer Res.*, **19**, 127.
MCCOY, G. W.—(1909) *J. med. Res.*, **21**, 285.
MCKENNY—(1906) *Vet. J.*, **62**, 643.
MALINZIEWICZ, C. AND KRAMARZ, K.—(1949) *Med. Weteryn.*, **5**, 56.
MAMMOLI, R.—(1959) *Ann. Fac. Med. vét. Pisa*, **11**, 1.
MARIE, P. AND AUBERTIN, C.—(1911) *Bull. Ass. franç. Cancer*, **4**, 253.
MEIER, H.—(1956) *Cornell Vet.*, **46**, 188.
MEISSNER, W. A., SOMMERS, S. C. AND SHERMAN, G.—(1957) *Cancer*, **10**, 500.

MIGLIAVACCA, L.—(1950) *Clin. vet., Milano*, **73**, 171.
MONLUX, A. W., ANDERSON, W. A. AND DAVIS, C. L.—(1956) *Amer. J. vet. Res.*, **17**, 646.
*Iidem* AND MONLUX, W. S.—(1956) *Ibid.*, **17**, 45.
MONTPELLIER, J. M., SAMSO, A. AND CATANEI, J.—(1952) *Bull. algèr. Cancer*, **5**, 445.
MÜLLER, F.—(1954) *Berl. Münch. tierärztl. Wschr.*, **67**, 240.
MURRAY, J. A.—(1908) *Sci. Rep. Cancer Res. Fd, Lond.*, **3**, 41.
NEWBERNE, J. W. AND ROBINSON, V. B.—(1960) *Amer. J. vet. Res.*, **21**, 150.
ORR, J. W. AND POLSON, C. J.—(1938) *Amer. J. Cancer*, **32**, 114.
OTTOSEN, H. E.—(1943) *Skand. VetTidskr.*, **33**, 473.
OVERGOOR, G. H. A.—(1958) *Tijdschr. Diergeneesk.*, **81**, 436.
PAINE, R.—(1909) *J. comp. Path.*, **22**, 349.
PIERSON, H.—(1935) *Z. Krebsforsch.*, **41**, 103.—(1937*a*) *Ibid.*, **45**, 1.—(1937*b*) *Ibid.*, **46**, 109.—(1938*a*) *Ibid.*, **47**, 1.—(1938*b*) *Ibid.*, **47**, 336.
PLAUT, A. AND KOHN-SPEYER, A. C.—(1947) *Science*, **105**, 391.
POLLIA, J. A.—(1938) *Amer. J. Cancer*, **32**, 545.
POLSON, C. J.—(1927) *J. Path. Bact.*, **30**, 603.
RATCLIFFE, H. L.—(1932) *Amer. J. Path.*, **8**, 117.—(1933) *Amer. J. Cancer*, **17**, 116.—(1940) *Amer. J. Path.*, **16**, 237.
REWELL, R. E. AND WILLIS, R. A.—(1949) *J. Path. Bact.*, **61**, 454.
RISER, W. H.—(1940) *J. Amer. vet. med. Ass.*, **96**, 271.—(1942) *Ibid.*, **100**, 65.
RIVIÈRE, M. R., CHOUROULINKOV, I. AND GUÉRIN, M.—(1960) *Bull. Ass. franç. Cancer*, **47**, 542.
RUSK, G. Y. AND EPSTEIN, N.—(1927) *Amer. J. Path.*, **3**, 235.
SCHLOTTHAUER, C. F.—(1939) *J. Amer. vet. med. Ass.*, **95**, 181.
SCHOLER, P. T.—(1916) *Z. Krebsforsch.*, **15**, 193.
SCHULZE, F.—(1960) *Ibid.*, **64**, 78.
SCHURMANN, E. A.—(1915) *J. comp. Path.*, **27**, 330.
SEDLMEIER, H.—(1930) *Münch. tierärztl. Wschr.*, **81**, 89.
SEGOLINI, A. B.—(1930) *Clin. vet. Milano*, **53**, 67.
SELINOW—(1908) Abstract in *Zbl. allg. Path. path.*, **19**, 122.
SHATTOCK, S. G.—(1900) *Trans. path. Soc. Lond.*, **51**, 56.
SIGNOL, J.—(1951) *Rev. Méd. vét.*, **102**, 567.
SINGER, C.—(1913–14) *J. Path. Bact.*, **18**, 495.
SLYE, M., HOLMES, H. F. AND WELLS, H. G.—(1924) *J. Cancer Res.*, **8**, 96.
SNELL, G. D. (Ed.)—(1956) 'Biology of the Laboratory Mouse.' New York (Dover Publications Inc.) pp. 225–227.
SOLDATI, M.—(1929) *Nuova vet.*, **7**, 292.
SOMMERS, S. C. AND MEISSNER, W. A.—(1957) *Cancer*, **10**, 510.
STERNBERG, S. S.—(1961) *Amer. J. Obstet. Gynec.*, **82**, 96.
STICKER, A.—(1902) *Arch. klin. Chir.*, **65**, 616, 1023.
STILLING, H. AND BEITZKE, H.—(1913) *Virchows Arch.*, **214**, 358.
STRONG, L. C.—(1938) *Amer J. Cancer*, **32**, 80.
TAMASCHKE, C.—(1951–52) *Wiss. Z. Humboldt Univ. Berlin* (*Math. Naturwiss.*), **1**, 37.—(1955) *Strahlentherapie*, **96**, 150.
TEUTSCHLAENDER, O.—(1920) *Z. Krebsforsch.*, **17**, 284.
THIEULIN, M.—(1930) *Bull. Ass. franç. Cancer*, **19**, 820.
THOMPSON, S. W., HUSEBY, R. A., FOX, M. A., DAVIS, C. L. AND HUNT, R. D.—(1961) *J. nat. Cancer Inst.*, **27**, 1037.
THRASHER, J. P.—(1961) *J. Amer. vet. med. Ass.*, **138**, 27.
TROTTER, A. M.—(1906) *J. comp. Path.*, **19**, 41.—(1911) *Ibid.*, **24**, 1.
TWORT, C. C.—(1937) *J. Path. Bact.*, **44**, 492.
UDALL, D. H., FINCHER, M. G. AND CUSHING, E. R.—(1926) *Cornell Vet.*, **16**, 230.
USAWA, T.—(1930) *Sei-i-Kwai med. J.*, **49**, 5.
VANDEPLASSCHE, M. AND THOONEN, J.—(1950) *Vlaam. diergeneesk.Tijdschr.*, **19**, 214.

VERARDINI, G.—(1937) *Nuova Vet.*, **15**, 282.
WAGNER, G. A.—(1905) *Zbl. allg. Path. path.*, **16**, 131.
WARGNIEZ, M.—(1953) *Rec. Méd. vét.*, **129**, 16.
WATRIN, J. AND FLORENTIN, P.—(1930) *C.R. Soc. Biol., Paris*, **104**, 1286.
WILSON, G. S. AND MILES, A. A. (ed.)—(1955) in Topley and Wilson's ' Principles of Bacteriology and Immunity.' London (Edward Arnold), Vol. 1, p. 490.
WITHERSPOON, J. T.—(1938) *Amer. J. Cancer*, **33**, 389.
WOGLOM, W. H.—(1919) *Proc. N.Y. path. Soc.*, **19**, 60.
WYSSMANN, E.—(1912) *Schweiz. Arch. Tierheilk.*, **54**, 8.
ZEBRACKI, A.—(1962) *Wien. tierärztl. Mschr.*, **49**, 135.